# Conformal prediction enables disease course prediction and allows individualized diagnostic uncertainty in multiple sclerosis

**DOI:** 10.1038/s41746-025-01616-z

**Published:** 2025-04-24

**Authors:** Akshai Parakkal Sreenivasan, Aina Vaivade, Yassine Noui, Payam Emami Khoonsari, Joachim Burman, Ola Spjuth, Kim Kultima

**Affiliations:** 1 Department of Medical Sciences, Uppsala University, Uppsala, 75185 Sweden; 2 Department of Biochemistry and Biophysics, National Bioinformatics Infrastructure Sweden, Science for Life Laboratory, Stockholm University, Solna, 17121 Sweden; 3 Department of Pharmaceutical Biosciences and Science for Life Laboratory, Uppsala University, Uppsala, 75124 Sweden

**Keywords:** Multiple sclerosis, Machine learning

## Abstract

Accurate assessment of progression and disease course in multiple sclerosis (MS) is vital for timely and appropriate clinical intervention. The gradual transition from relapsing-remitting MS (RRMS) to secondary progressive MS (SPMS) is often diagnosed retrospectively with a typical delay of three years. To address this diagnostic delay, we developed a predictive model that uses electronic health records to distinguish between RRMS and SPMS at each individual visit. To enable reliable predictions, conformal prediction was implemented at the individual patient level with a confidence of 93%. Our model accurately predicted the change in diagnosis from RRMS to SPMS for patients who transitioned during the study period. Additionally, we identified new patients who, with high probability, are in the transition phase but have not yet received a clinical diagnosis. Our methodology aids in monitoring MS progression and proactively identifying transitioning patients. An anonymized model is available at https://msp-tracker.serve.scilifelab.se/.

## Introduction

Multiple sclerosis (MS) is an inflammatory, neurodegenerative disease affecting the central nervous system. It is a leading cause of neurological disability in young adults globally. The course of MS is heterogeneous but typically involves an early, predominantly inflammatory disease phase termed relapsing-remitting MS (RRMS) and a later, principally degenerative stage known as secondary progressive MS (SPMS). SPMS is diagnosed retrospectively, where the average delay is 3 years^1^. While current disease-modifying therapies are effective in RRMS, the majority have very limited benefit in SPMS, if at all. Proactive recognition of patients with progressive disease could limit exposure to ineffective medications and their side effects. Early identification of patients eventually fulfilling the criteria of SPMS would, therefore, be a valuable addition to the armamentarium of clinical practitioners, enabling meaningful intervention.

Previous studies have explored invasive and non-invasive biomarkers, including biochemical and imaging-based measures, for predicting disease progression^2,3^, and the transition to SPMS^4–11^. However, the predictive value of these markers is limited^4,12^, they lack an uncertainty measure, and they are not routinely used in clinical practice. One potential approach to timely disease progression identification is using artificial intelligence (AI) and machine learning (ML). Progress in these fields has opened up the possibility of assimilating and interpreting complex data in healthcare and is expected to be transformational^13^. Machine learning and deep learning (DL)-based methods have been developed to predict the transition from RRMS to SPMS^14^.

In a study by Manouchehrinia et al., the authors achieved an accuracy of 77–87% when predicting the risk of conversion to SPMS in 10, 15, and 20 years using a nomogram-based method^15^. The study used electronic health record data (EHR) from 8825 RR onset MS patients in Sweden and was validated using 6498 patients. However, the model was developed only using data from the first hospital visit from a certain patient, and there were no risk scores associated with each hospital visit. A similar study to predict transition to SPMS within 180, 360, or 720 days was carried out by Seccia et al., utilizing 1624 patients with 18,574 clinical records^14^. The tool was designed to make predictions using both historical clinical records and individual follow-ups. While this study demonstrated higher specificity and recall, the precision of the predictions was lower, resulting in an increased number of false positives being included. Both studies focused solely on RRMS patients, potentially missing those who had transitioned. Additionally, the studies did not incorporate any uncertainty measure for their predictions, making the model susceptible to errors when applied to external data.

As the transition from RRMS to SPMS is gradual, with overlapping disease processes during this transitional period, developing a binary classifier is challenging^16^. More generally, the adoption of predictive AI tools in healthcare thus far has been limited by more than solely their measured performance. Significant shortcomings in the clinical setting include an inability to convey uncertainty in a given prediction^17^ and a lack of explainability or interpretability for a given prediction^18^. The explainable AI (XAI) models can help healthcare practitioners understand and more easily verify the results provided by these models.

Conformal prediction (CP) is a framework for complementing single-valued predictions from standard ML/AI classifiers with a valid measure of the prediction’s uncertainty^19^. At a specified confidence level, the conformal predictor will provide a region around the point prediction containing the true label. For instance, when predicting a patient’s RRMS or SPMS disease state, CP produces four outputs: {RRMS}, {SPMS}, {RRMS, SPMS}, and {}. If the CP output contains multiple labels, the prediction incorporates more than one true label, thus predicting a patient to be both RRMS and SPMS. Conversely, if a CP generates empty predictions, it signifies that a valid prediction cannot be made. We have recently demonstrated that CP can substantially reduce the number of errors made by an AI classifier in grading prostate biopsies^20^ and that ML in combination with CP can aid in predicting the transition of SPMS based on biomarkers measured in cerebrospinal fluid (CSF) analysis^21^. However, this approach has not been assessed with EHR data alone, which could circumvent the need for invasive or costly biomarkers.

In this study, we develop conformal predictors for ML-assisted diagnostics in MS using clinical information from the EHR collected from 22,748 MS patients with 197,227 hospital visits. We demonstrate that the model is well-calibrated, meaning the conformal predictors are valid. This allows us to produce reliable predictive uncertainties for each patient’s hospital visit. We also show how these predictors can be used to monitor a patient’s disease progression in the spectrum between RRMS and SPMS, allowing earlier identification of patients fulfilling the criteria of SPMS. We then incorporated SHapley Additive exPlanations (SHAP) to demonstrate the contributions of clinical variables to the individual predictions and the entire test dataset^22^.

We believe this approach can assist in monitoring the disease progression, earlier identification of transition to SPMS, and provide a powerful tool for tracking interventions’ effects that can also be used in clinical trials. Finally, we have set up a publicly accessible web server deploying the ML architecture for research use only.

## Results

We trained an AI model to identify patients with diagnoses of either RRMS or SPMS using EHR data from the Swedish MS Registry (SMSReg)^23^. The SMSReg is a nationwide registry containing data from 22,748 MS patients, with 197,227 hospital visits, collected between 1972 and 2022. The registry has high validity and broad coverage, estimated to include over 80% of all people with MS in Sweden^24^. More than 850 neurologists have contributed data to the registry.

**Fig. 1:**
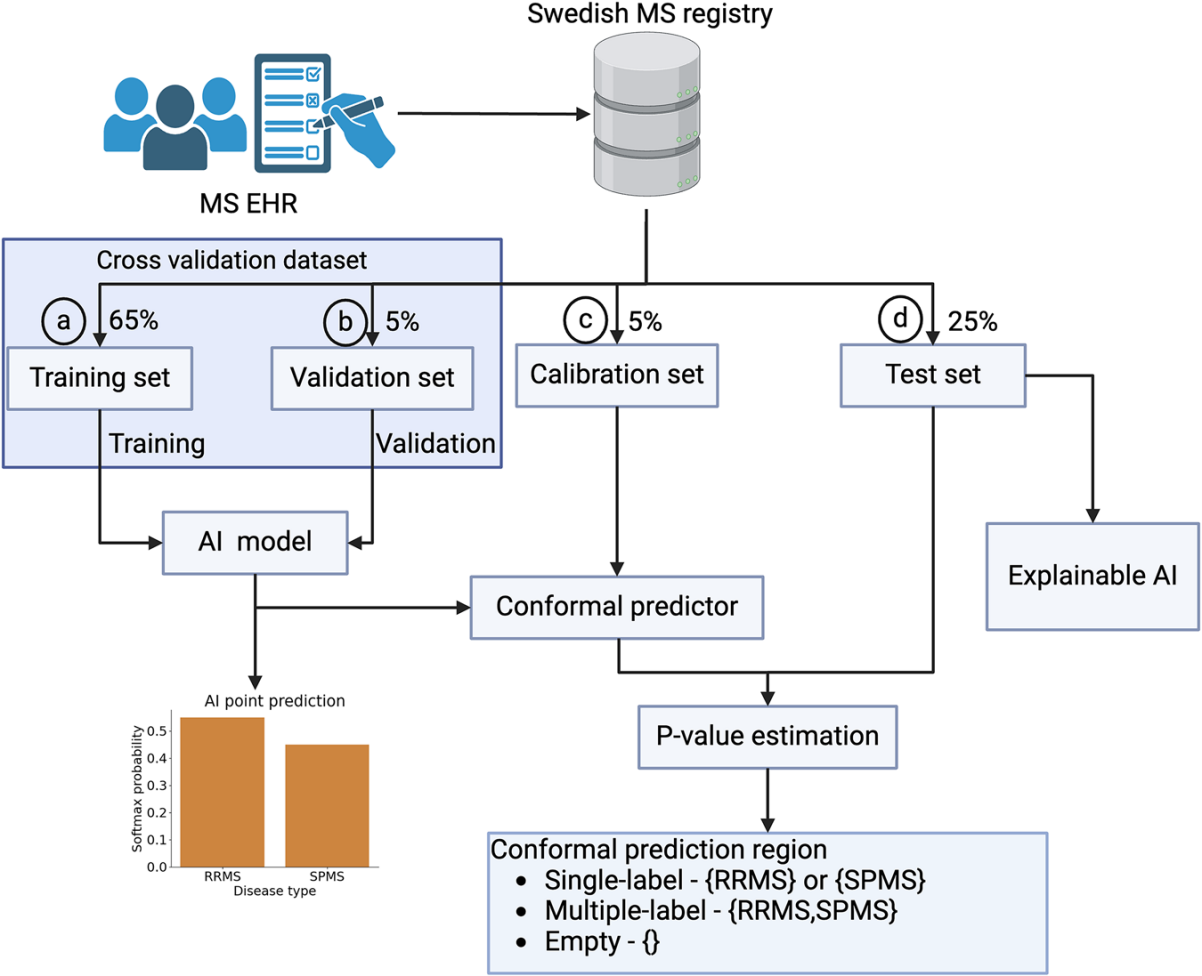
Overview of model training, validation, calibration, and testing*.* **a** 65% of the data was used for training, with **b** 5% used as a validation set for the validation. **c** 5% of the data was kept aside as the calibration set **d** 25% was set aside as the test set to evaluate the model efficiency. Created with BioRender.com. Kultima, K. (2024) https://BioRender.com/t48p033.

The data from the registry was processed as illustrated in Fig. 1. Only patients with an RRMS or SPMS diagnosis at the first presentation were included. Duplicate entries were removed, and individual patient records were divided into hospital visits. Fifty-six clinical parameters from the EHR were used to generate 61 derived features (Supplementary Table 1). The dataset was split into four non-overlapping subsets of patients for the training (individual patients, np = 9348), validation (np = 719), calibration (np = 720), and testing (np = 3595) of the models. The baseline characteristics of the patients in these four subsets were similar, as outlined in Table 1.

**Table 1:** Patient characteristics in the train, valid, calibration, and test dataset

	Train dataset np = 9348; nv = 74,064		Valid dataset np = 719; nv = 5626		Calibration dataset np = 720; nv = 5771		Test dataset np=3595; nv = 28,323	
Features	RRMS (np = 6414: nv = 48,393)	SPMS^a^ (np = 2934: nv = 25,671)	RRMS (np = 491: nv = 3677)	SPMS^b^ (np= = 228: nv = 1949)	RRMS (np = 467: nv = 3657)	SPMS^c^ (np = 253: nv = 2114)	RRMS (np = 2451: nv = 18,525)	SPMS^d^ (np = 1144: nv = 9798)
Female %	71.9	71.0	70.1	66.7	73.0	68.0	72.8	69.3
Age ± SD	40.4 ± 10.9	56.3 ± 10.8	39.9 ± 10.7	55.4 ± 10.5	40.8 ± 11.0	56.7 ± 10.8	40.6 ± 11.1	55.6 ± 10.6
EDSS ± SD	1.7 ± 1.2	5.6 ± 1.7	1.7 ± 1.2	5.4 ± 1.7	1.8 ± 1.2	5.4 ± 1.6	1.8 ± 1.2	5.7 ± 1.7
Disease duration ± SD	8.7 ± 7.0	21.9 ± 11.3	8.8 ± 7.2	21.2 ± 12.0	8.9 ± 6.9	21.5 ± 11.8	9.0 ± 7.4	21.8 ± 11.0
Debut relapse age ± SD	23.9 ± 16.8	27.1 ± 17.2	23.9 ± 16.3	26.1 ± 17.8	24.7 ± 16.3	28.1 ± 17.8	24.2 ± 16.8	25.5 ± 17.1

Distributions of patients with diagnoses of RRMS and SPMS. Distributions are similar in all datasets (train, valid, calibration, and test). In this table, patients recorded to have a diagnosis of RRMS at the final hospital visit were categorized as RRMS. Patients with a diagnosis of SPMS were categorized as SPMS. In the SPMS patients, patients with SPMS at initial and final hospital visits (SPMS-SPMS) ^a^np = 1696; nv = 10,542, ^b^np = 125; nv = 813, ^c^np = 152; nv = 904, ^d^np = 677; nv = 3977 and patients with RRMS at initial and SPMS at final hospital visits (RRMS-SPMS) ^a^np = 1238; nv = 15,129, ^b^np = 103; nv = 1136, ^c^np = 101; nv = 1210, ^d^np = 467; nv = 5821. np = number of individual patients, nv = number of hospital visits.

To account for uncertainty on an individual patient level, we used CP and assessed the model efficiency as the fraction of all the predictions, resulting in a single-label prediction. We also evaluated the model’s validity, i.e., the error rate did not exceed the pre-specified significance level of the conformal predictor, added XAI using SHAP to elucidate the features influencing the predictions, and developed a publicly available model for use in research.

### Machine learning models on EHR data produce accurate models to predict RRMS and SPMS

First, we assessed the performance of different ML models in predicting whether a patient had a diagnosis of RRMS or SPMS at a given hospital visit. Four ML models were trained: logistic regression, support vector machines (SVM), gradient-boosting (GB), random forest (RF), and a DL model (‘long short-term memory’, LSTM). The latter was selected for its ability to use historical information from prior hospital visits to guide predictions for the same patient in subsequent visits.

We evaluated the ML and DL models using 10-fold cross-validation on the combined training and validation datasets (individual patients, np = 10,067; hospital visits, nv = 79,690). Based on the macro average F1 score, the combined measure of precision and recall, the performance in discriminating between RRMS and SPMS at hospital visits was high. RF, SVM, and GB all had an F1 score of 0.90. These three models significantly outperformed logistic regression and LSTM (*p* < 0.05, Supplementary Figs. 1 and 2). Since the three traditional ML models performed similarly to one another, we selected RF (0.903 ± 0.008) for subsequent analysis.

In many cases, the information the different clinical variables hold is redundant. We investigated whether we could identify a minimal number of clinical features used in the model without negatively impacting the overall performance. On average, the RF model performed best when we excluded the information from the patient-reported multiple sclerosis impact scale (MSIS-29), retaining 27 features (Supplementary Table 1, and Supplementary Figs. 3 and 4).

### Conformal prediction produces valid and efficient models for predicting MS diagnosis at a hospital visit

**Fig. 2:**
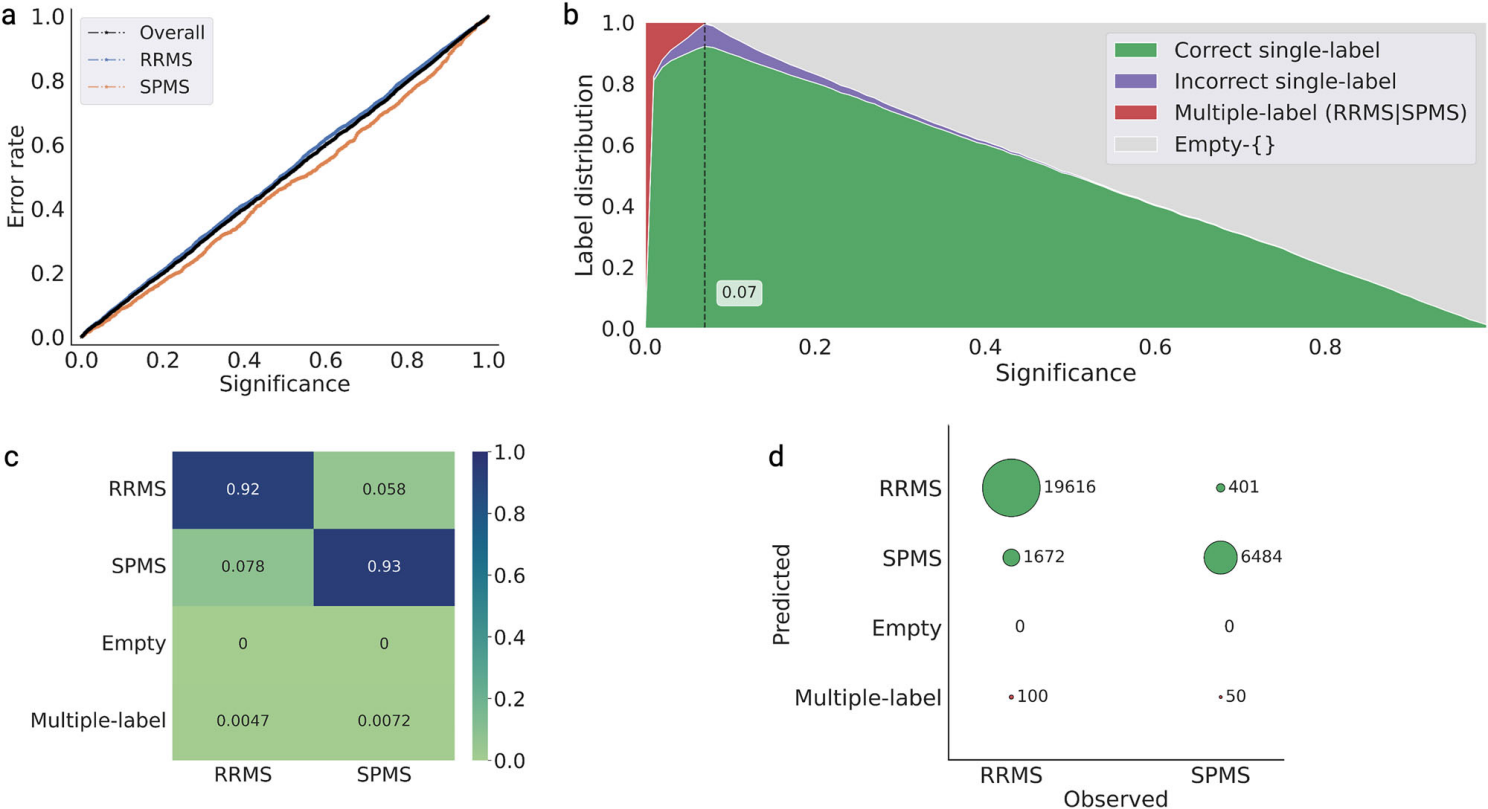
Calibration and efficiency plots on the test set data. **a** The calibration plot shows the observed prediction error on the *y*-axis and the prespecified significance level on the *x*-axis, i.e., the tolerated error rate. The observed error rate is close to the diagonal line, indicating a valid conformal predictor. **b** The efficiency plot shows the label distribution of correct single-label, incorrect single-label, multiple-label, and empty predictions for the test set at different significant levels. The plot demonstrates the expected confidence-efficiency trade-off, whereby lower significance levels (higher confidence levels) result in the conformal predictor returning an increasing proportion of multiple-label prediction and vice versa, returning an increased proportion of empty prediction at lower confidence. The confidence level corresponds to a 1-significance level. The peak single-label prediction (i.e., the highest proportion of single-label predictions) is at 93% confidence, corresponding to a significance of 0.07. **c** Normalized predictions in the test set data at 93% confidence (highest efficiency) with the predictions RRMS and SPMS indicate single-label prediction, whereas empty represents no prediction, and multiple-label represents both RRMS and SPMS prediction. **d** Bubble plot showing prediction of the test set at 93% confidence.

We added a valid measure of the prediction uncertainty using CP to complement the single-valued prediction from the best-performing RF model. The output *p*-values from the model were calibrated using the calibration dataset with data from 720 patients at 5771 hospital visits. While in traditional statistics, the *p*-value suggests evidence against a null hypothesis, the *p*-values in this context measure how well the current observation conforms to the previously observed data i.e., calibration dataset. The calibration plot (Fig. 2a) demonstrates the very close correspondence between the specified significance level and the resulting observed prediction error, indicating excellent validity of the conformal predictor.

The performance of the conformal predictor can be illustrated at different pre-specified significance levels (Fig. 2b). The conformal predictor had the highest proportion of correct single-label predictions at a significance level of 0.07, i.e., a confidence level of 93%. Consequently, we evaluated the efficiency of the conformal predictor at a 93% confidence level for predicting RRMS or SPMS in the test dataset.

### Prediction with confidence for all the hospital visits using conformal prediction

The test dataset contained 3595 patients and 28,323 hospital visits. Of these, 2451 patients (18,525 hospital visits) were diagnosed with RRMS throughout, and 677 with SPMS (3977 hospital visits). The remaining 467 patients (5821 hospital visits) had a diagnosis of RRMS at the first visit and a diagnosis of SPMS at the last hospital visit. Based on these data, we evaluated the conformal predictor’s ability to determine the correct diagnosis (i) at each hospital visit, (ii) the final diagnosis for each patient, and (iii) in patients with an initial diagnosis of RRMS and a final diagnosis of SPMS (“transitioning” patients). The final group was also evaluated based on the visit at which the patient was first diagnosed with SPMS relative to the conformal predictor's initial prediction of SPMS for each patient.

When analyzing each hospital visit, the proportion of correct single-label predictions was high (92.1%). There were a total of 2073 (7.3% of test set data) incorrect single-label predictions, with no instances of empty predictions and 150 (0.5% of test set data) instances of multiple-label predictions (RRMS | SPMS) (Fig. 2c, d). From the incorrect predictions, in 1672 cases (7.8% of all RRMS hospital visits), the patients were erroneously predicted as having SPMS, and in 401 cases (5.8% of all SPMS hospital visits), RRMS. Since the course of MS is heterogeneous with periods of relapses, it is not unexpected that at some hospital visits, there will be incorrect predictions. However, a closer inspection of these errors reveals that 56.8% of the erroneous SPMS predictions originated from only 118 patients (3.3% of all MS patients in the test set). Similarly, 54.9% of the incorrect RRMS predictions originated from only 40 patients (1.1% of all MS patients in the test set) (Supplementary Fig. 5).

**Fig. 3:**
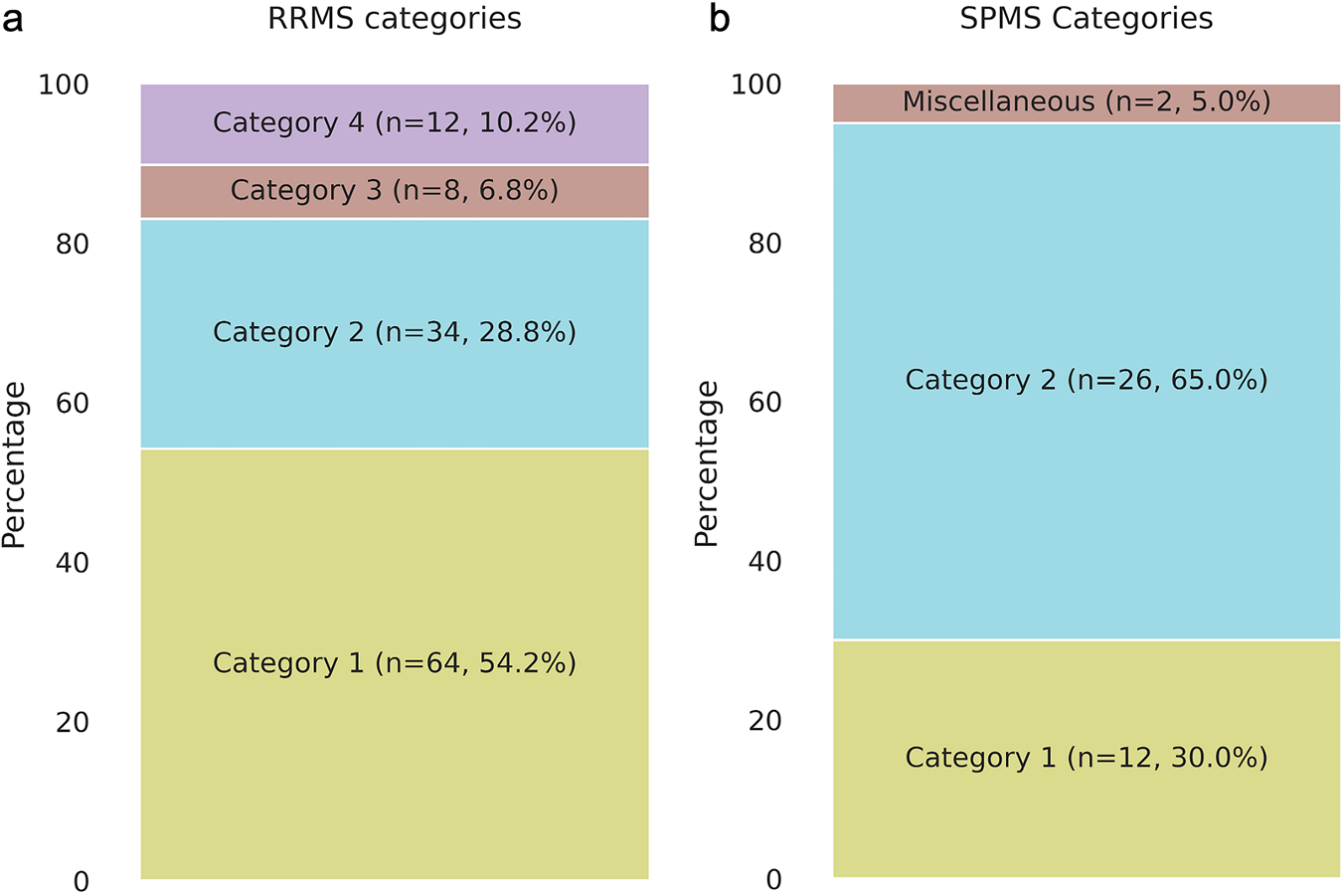
Deeper analysis of frequently misclassified patients’ predictions. The patients were categorized as follows: **a** 118 frequently misclassified RRMS patients grouped into four categories, and **b** 40 frequently misclassified SPMS patients grouped into three categories.

We conducted a deeper analysis of these frequently misclassified patients, examining their clinical characteristics in more detail. To better understand these erroneous predictions, we manually classified them into distinct categories based on how their CP *p*-values evolved over time. The 118 frequently misclassified RRMS patients (with erroneous SPMS predictions) were grouped into four categories—category 1, 2, 3, and 4—while the 40 frequently misclassified SPMS patients (with erroneous RRMS predictions) were grouped into three categories—category 1, 2, and miscellaneous (Fig. 3).

**Fig. 4:**
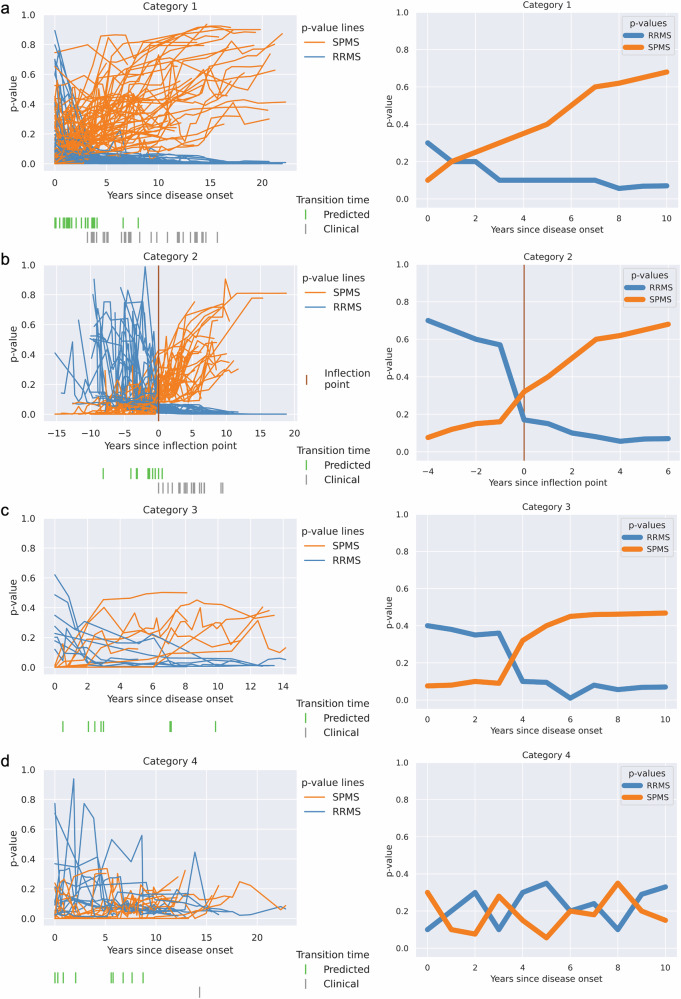
Four distinct categories for the 118 frequently misclassified RRMS patients. The *p*-values for the RRMS and the SPMS from the CP were plotted (on the left side), and the corresponding schema of how the patients were categorized (on the right side). **a** Categorized based on the presence of increasing SPMS *p*-values from the beginning of their earliest recorded clinical visits. **b** An inflection point marked by a rapid increase in SPMS *p*-values was identified and established as the baseline time point. This category resembles Category 1 but with additional prior clinical data. **c** Category where high EDSS score led to incorrect SPMS predictions, despite stable clinical course. **d** The model’s predictions switch between disease states along the duration of the disease course, highlighting inaccurate predictions.

**Fig. 5:**
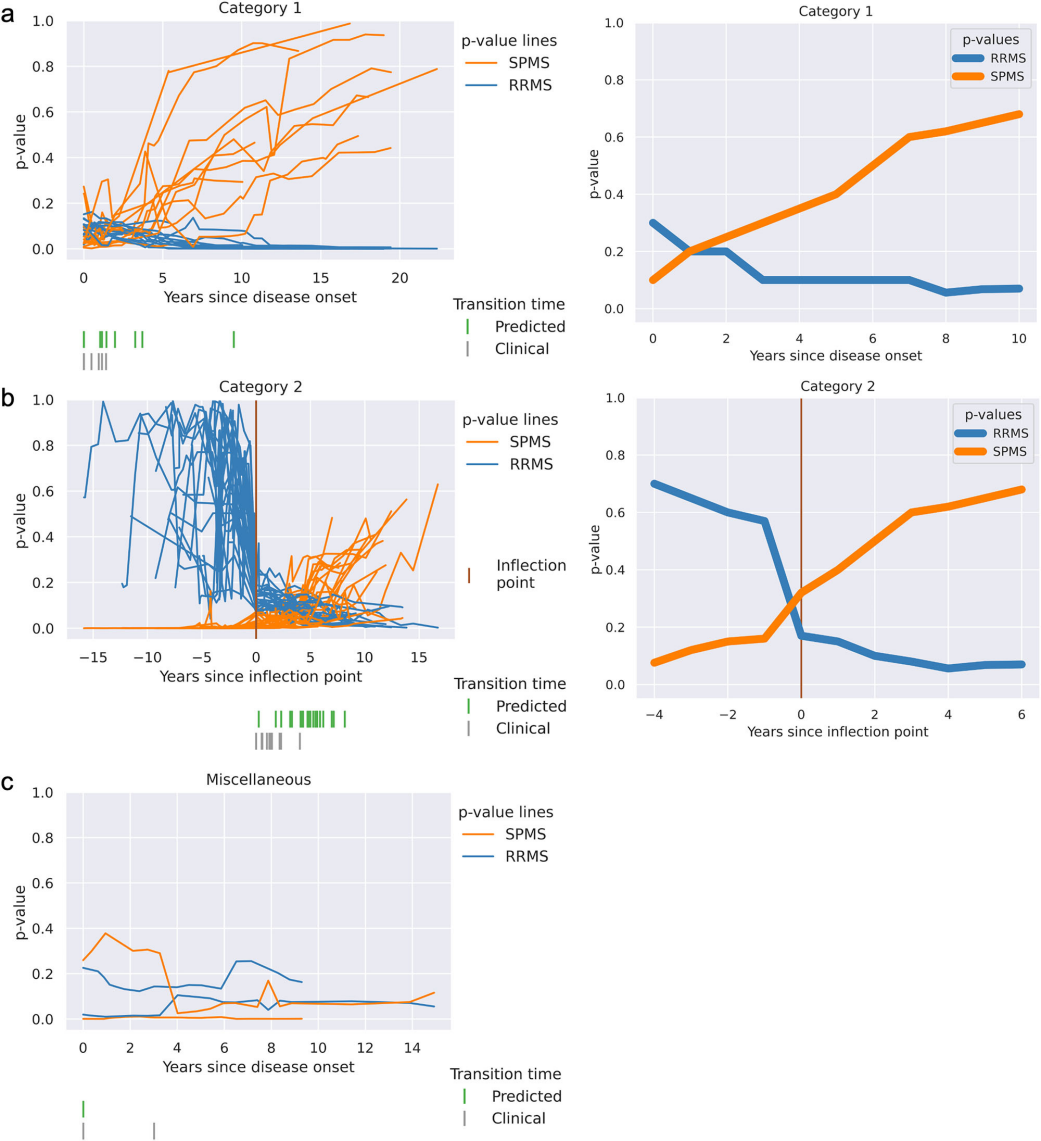
Three distinct categories for the 40 frequently misclassified SPMS patients. The *p*-values for the RRMS and the SPMS from the CP were plotted (on the left side), and the corresponding schema of how the patients were categorized (on the right side). **a** Categorized based on the presence of increasing SPMS *p*-values from the beginning of their earliest recorded clinical visits. **b** An inflection point marked by a rapid decrease in RRMS p-values was identified and established as the baseline time point. This category resembles Category 1 but with additional prior clinical data. **c** Category in which no clear pattern emerged from our analysis.

The patterns we describe for categories 1 and 2 were present in both the frequently misclassified RRMS patients and the frequently misclassified SPMS patients (Figs. 4a, b and 5a, b). Category 1 was characterized by steadily increasing SPMS p-values from the first hospital visit onwards, with low RRMS *p*-values throughout (Figs. 4a and 5a). Category 2 was characterized by a low SPMS *p*-value for a variable length of time, followed by a steady increase in the SPMS *p*-value (Figs. 4b and 5b). We found several commonalities between clinical features when we categorized patients into these categories based on their SPMS and RRMS *p*-values over time. Both categories 1 and 2 appeared to represent similar trends, with the difference that patients in category 2 had more visit information early on in the disease process (as suggested by the period of low SPMS *p*-values). This could be seen when the trends were plotted with the 'inflection point' in *p*-values—the point at which the SPMS *p*-values started to increase, or RRMS *p*-values decrease were aligned in time (Figs. 4b and 5b).

When only considering the frequently misclassified RRMS patients, it can be noted that within categories 1 and 2, the model consistently predicted these patients to have SPMS earlier than the diagnosis recorded by the registry data (Fig. 4a, b) and may potentially represent a diagnostic delay.

Conversely, for the frequently misclassified SPMS patients in categories 1 and 2, the model identified transitions to SPMS at a later time point than that recorded in the registry. In category 2, we observed a rapid decrease in RRMS probability at the time point where the transition from RRMS to SPMS was identified in the registry. The patients in these categories were generally younger adults (<40 years old) and/or those with lower EDSS scores (<4.5), which are less typical for disease transition. The model failed to identify the correct disease state at these transition points.

Category 3 contained only frequently misclassified RRMS patients, with SPMS p-values that initially increased but soon plateaued (Fig. 4c). This typically consisted of patients with a diagnosis of RRMS throughout all visits, but who also had high EDSS scores, which we found to be due to poor recovery of disability following relapses rather than progressive disease. Category 4 also only contained frequently misclassified RRMS patients, with the model’s classification fluctuating between RRMS and SPMS (Fig. 4d). Thus, categories 3 and 4 clearly represented erroneous predictions, where the model performs poorly in comparison to registry data.

Taken together, looking at the most frequently misclassified patients, we noted that for misclassified RRMS patients within categories 1 and 2 (83.1% of misclassified RRMS), the model identified transition earlier than recorded in the registry, whereas the misclassified SPMS patients in categories 1 and 2 (95% of misclassified SPMS), the model identified disease transition later than the registry. This may be due to less typical clinical features in these patients. In categories 3 and 4 (16.9% of misclassified RRMS), the model performed worse compared to the diagnoses recorded in the registry, while some patients (‘miscellaneous’ category, 5% of misclassified SPMS, Figs. 3 and 5c) did not fit into any of these categories.

To identify the number of patients contributing to 50% of the erroneous predictions in the entire dataset, a 4-fold cross-validation (CV) was performed by keeping 70% training, 5% calibration, and 25% testing data. The analysis revealed that approximately 50% of the erroneous SPMS predictions originated from a small subset of 405 (2.8% of all MS patients in the study) patients. Similarly, 147 (1% of all MS patients in the study) patients accounted for about 50% of all erroneous RRMS predictions. These results indicate that the correct single-label prediction efficiency of the conformal predictor is high, and the erroneous predictions often originate from a smaller fraction of all patients.

### Conformal prediction enables efficient prediction of MS diagnosis on a patient level with individual confidence measures

As SPMS is diagnosed retrospectively, we sought to evaluate the conformal predictor's ability to predict clinical courses based on the latest available diagnosis. The overall efficiency for predicting the latest diagnosis was high (94.1%) (Table 2). There were no empty predictions, six RRMS patients and one SPMS patient received multiple-label predictions (RRMS | SPMS). At a confidence level of 93%, there were 206 erroneous predictions. 179 of these (7.3% of all RRMS patients) were patients with the latest diagnosis of RRMS who were instead predicted to have SPMS. The remaining 27 (2.4% of all SPMS patients) patients had a final diagnosis of SPMS and were predicted to have RRMS.

**Table 2:** Prediction at final hospital visits on the test dataset with a confidence of 93% compared to the clinical diagnosis

Conformal prediction with a confidence of 93%. Overall efficiency was 94.1%.		Clinical diagnosis	
		RRMS (np = 2451)	SPMS (np = 1144)
Prediction	RRMS	2266 (92.5%)	27 (2.4%)
	SPMS	179 (7.3%)	1116 (97.6%)
	Empty-{}	0 (0%)	0 (0%)
	Multiple-label (RRMS | SPMS)	6 (0.2%)	1 (0.1%)

The predictions RRMS and SPMS indicate single-label prediction, whereas empty represents no prediction, and multiple-label represents both RRMS and SPMS prediction for the hospital visit. *np* = number of individual patients. *Percentages would not add up due to rounding off.

Following the prediction efficiency being markedly asymmetrical (97.6% for SPMS, 92.4% for RRMS), we investigated the conformal predictor’s output *p*-values for the 179 patients incorrectly predicted to have SPMS. These cases could be grouped into three categories: a majority (111 patients, 4.5% of all RRMS patients) had predictions of RRMS at the initial visit, with predictions of SPMS at later visits, while the clinical diagnosis remained RRMS (Supplementary Fig. 6).

A group of patients (52 patients, 2.1% of all RRMS patients) persistently had predictions of SPMS at all visits despite diagnoses of RRMS, suggesting they could have SPMS already since their first presentation (Supplementary Fig. 7). And 16 patients had conflicting predictions with p-values suggesting uncertain predictions (Supplementary Fig. 8).

### Conformal prediction coupled with XAI enables the prediction of transition states from RRMS to SPMS diagnosis

We applied our model to predict the clinical course of transitioning patients. Given the retrospective nature of the SPMS diagnosis and previously demonstrated diagnostic delays, we assessed the conformal predictor’s performance in patients who “transitioned” from a clinical course of RRMS to SPMS between the first and last visit (np = 467, nv = 5821).

**Fig. 6:**
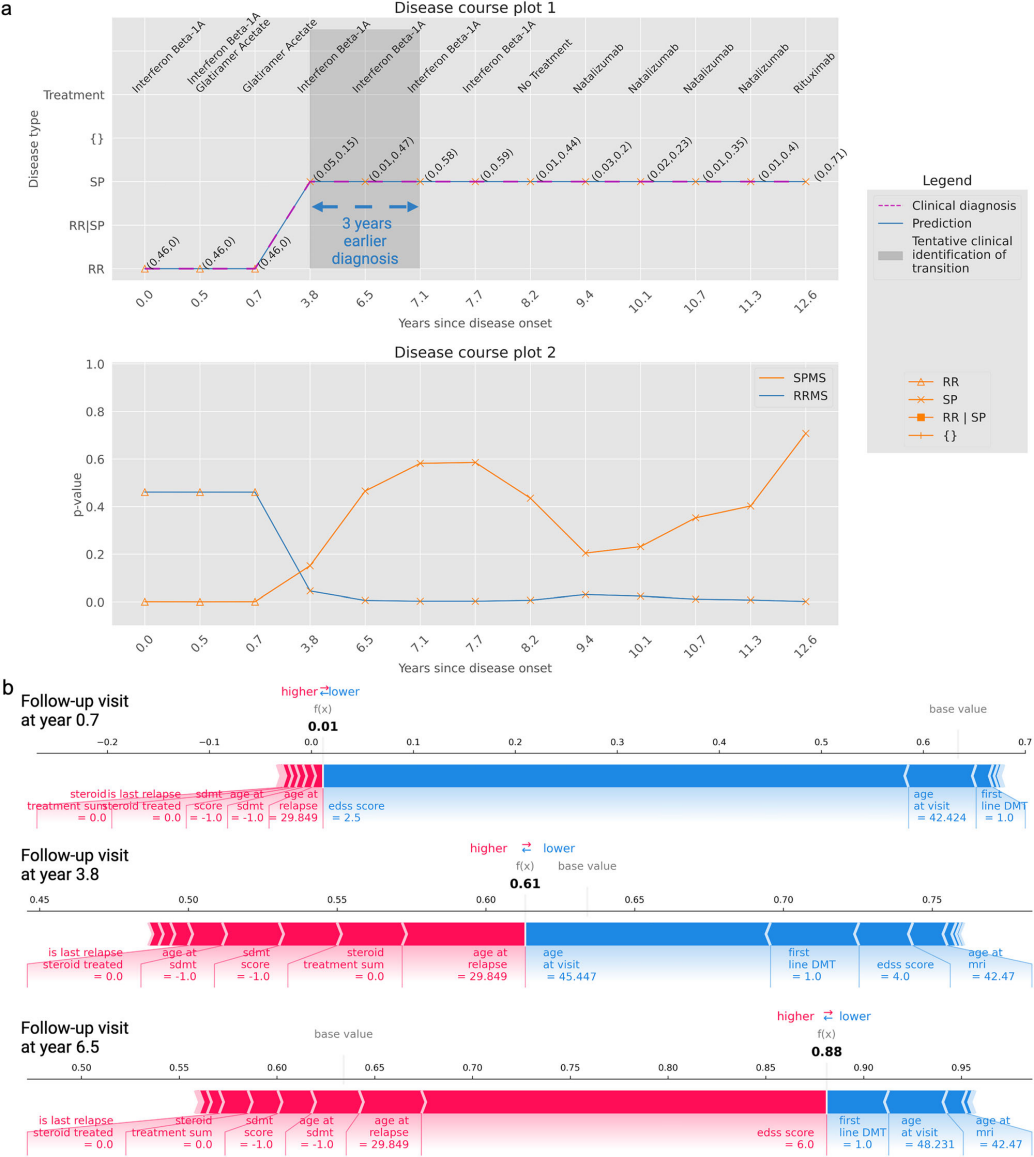
Predicted disease course at 93% confidence for an example patient with 13 hospital visits complemented with XAI. **a** Disease course of a transitioning patient with RRMS at the initial hospital visit and SPMS at the final hospital visit. In disease course plot 1 (top figure), the model predicts that the transition occurred in year 3.8. However, the clinic’s SPMS assessment could not be made until between year 3.8 and year 7.1. Thus, as indicated by the gray zone, a delay of three years is observed. Thus, the model identifies SPMS early, approximately three years in advance. Disease-modifying treatment names taken during the disease course are listed atop the figure. The disease course plot 2 (bottom figure) manifests the progression of the disease towards SPMS, indicating the disease worsening over time. A clear drop in RRMS *p*-value occurs between years 0.7 and 3.8, and at the same time, an increasing p-value score for SPMS is observed (between years 0.7 and 7.1). As the disability accumulates, the plot illustrates a decreasing RRMS *p*-value with an increasing SPMS *p*-value. **b** Feature contribution explanation using force plots for the predictions on the hospital visit at years 0.7, 3.8, and 6.5 of the patient. During the hospital visit year 0.7, the model predicted RRMS, driven by lower EDSS score, first-line DMT, and age at the visit. Meanwhile, the features contributing to SPMS are minimal. Conversely, in year 3.8, the model predicted SPMS, influenced by factors such as age at relapse, number of steroid treatments, age at SDMT, and lack of steroid treatment. Features such as age at visit, first-line DMT, EDSS score, and age at MRI contributed towards RRMS. By year 6.5, a high EDSS score considerably influenced the prediction of SPMS, while first-line DMT and age at visit contributed to RRMS. (The results of all visits are found in Supplementary Figs. 9–11).

Of 467 cases, the conformal predictor correctly predicted 320 (68.5%) to have RRMS at onset and later transition to SPMS (Supplementary Table 2, Fig. 6a for a patient example). In 125 cases (26.8%), the conformal predictor predicted that the patient had SPMS from disease onset. In 96 of these cases, they were predicted as having SPMS at all 812 subsequent hospital visits. The remaining 29 cases had subsequent multiple-label or incorrect RRMS predictions, followed by SPMS predictions. These results display high agreement between the diagnosis and predictions; 95.3% are correctly predicted to have SPMS, and when the two deviate, the conformal predictor typically predicts the patient as having SPMS from the first presentation.

To aid in understanding and verifying the predictions made at each hospital visit, the weightage given to features by the model can be interpreted using SHAP (Fig. 6b). For instance, EDSS scores strongly contribute to predictions towards RRMS at year 0.7 (EDSS 2.5) and SPMS at year 6.5 (EDSS 6.0). Factors such as first-line DMT and age at visits are strong indicators for RRMS, whereas factors such as lack of steroid treatments, age at relapse, etc., increase the likelihood of SPMS. However, at year 3.8, where the model predicts SPMS, the EDSS of 4.0 suggests RRMS, which would typically not be sufficient for an SPMS diagnosis. Instead, other factors like age at relapse, lack of steroid treatment, etc., collectively contribute to the SPMS prediction. With this in mind, SHAP analysis can be particularly useful in cases where the predictions from the model are discordant with the clinical diagnosis, or where a double-label prediction is made. For such cases, if the factors and the SHAP weights were available to clinicians, they could highlight patients who may be at higher risk of disease transition, as well as explain why such a prediction was made. Ultimately, this provides additional, contextualized information that could inform clinical decision-making.

Upon analysis of collective feature contribution using the entire test set data, the model demonstrates that EDSS and the age at the hospital visit had notable contributions compared to other features (Supplementary Fig. 12). The features SDMT score, age at debut relapse, age at MRI, first-line DMT, debut age, treatment, and age at SDMT had moderate contributions compared to the rest of the 18 features. This demonstrates the ability of CP and XAI to aid in early diagnosis, which also may assist in enabling meaningful intervention at an earlier stage.

### Predicting the timing of a change in diagnosis from RRMS to SPMS

**Fig. 7:**
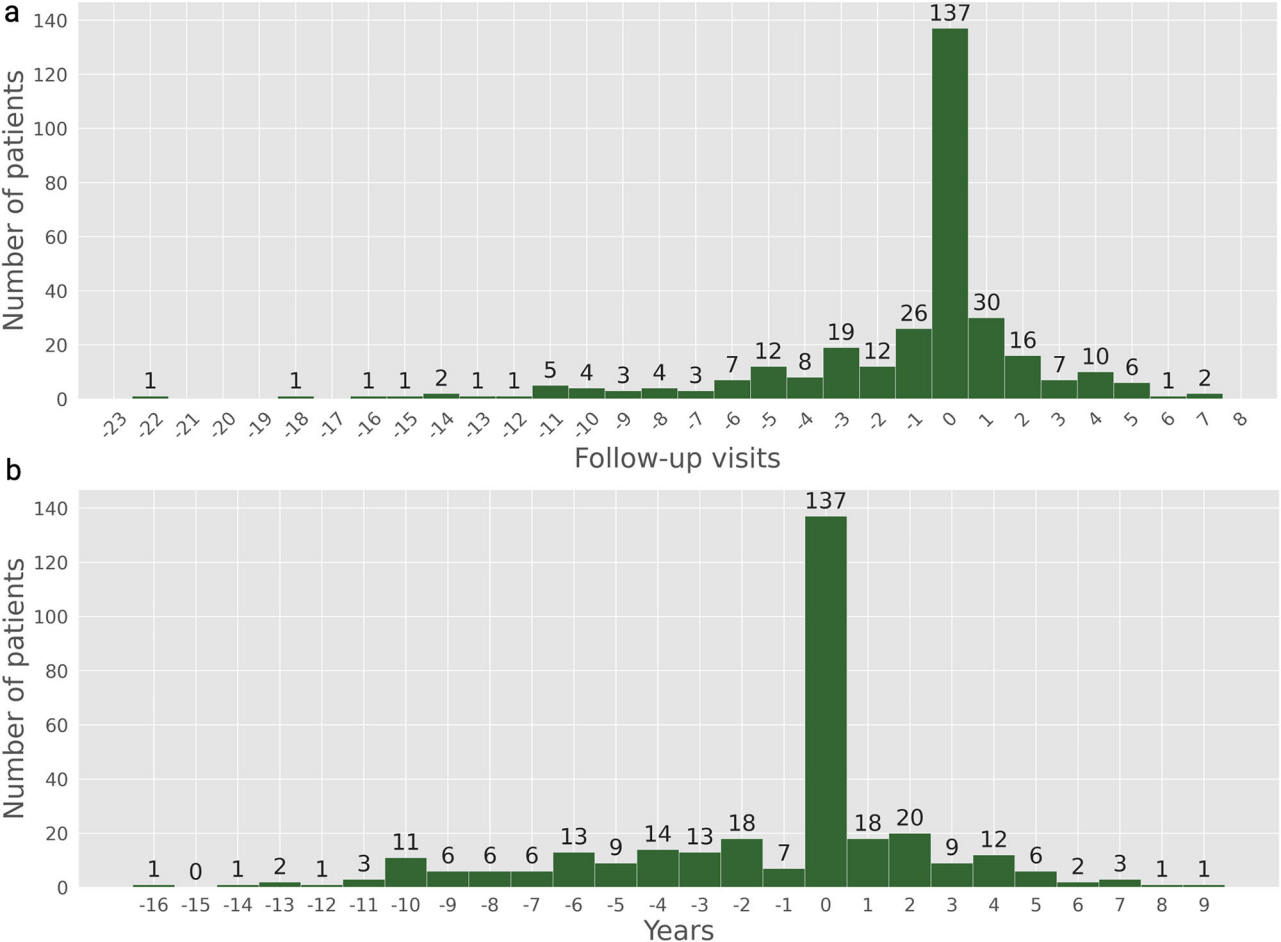
Difference of the time (hospital visits and years) for change from RRMS to SPMS diagnosis (320 patients) as found in EHR compared to the predictions at 93% confidence. An earlier prediction of SPMS is illustrated by negative values and vice-versa for a later SPMS prediction. **a** The difference in hospital visits. **b** The difference in years. Example: In **a**, the model predicted SPMS in 26 patients one visit earlier than the clinician did, and in 30 patients one visit later.

Next, we examined the concurrence between the time (hospital visit) when the patient was diagnosed with SPMS and the prediction made by the model at 93% confidence. In 320 cases that transitioned from RRMS to SPMS (Supplementary Table 2), there was a precise time point when the conformal predictor predicted a change in disease state. In 137 cases (42.8%), the time for a change in disease state was predicted the same as the clinician has set in retrospect (Fig. 7). In 56 cases (17.5%), it was just one hospital visit in difference. In the remaining 127 cases (39.7%), the conformal predictor predicted SPMS at an earlier hospital visit (85 cases, 26.6%) or a later hospital visit (42 cases, 13.1%). These results display a high degree of agreement with the time for a change in diagnosis from RRMS to SPMS and the prediction made by the model. In 86.9% of the cases, the predictions agreed with the time for a change in diagnosis within a deviation of one visit or predicted the time for change at an earlier time.

### Effects of increasing the confidence level in the conformal prediction for predicting diagnosis

In 23 out of the 320 cases where the patient changed their diagnosis from RRMS at debut to SPMS at the latest diagnosis, the predictions made by the conformal predictor were associated with a higher degree of uncertainty. This means that for at least two consecutive hospital visits with a period of >3 months between the visits, the patient was predicted SPMS but then changed back to RRMS, which is generally not considered possible.

**Fig. 8:**
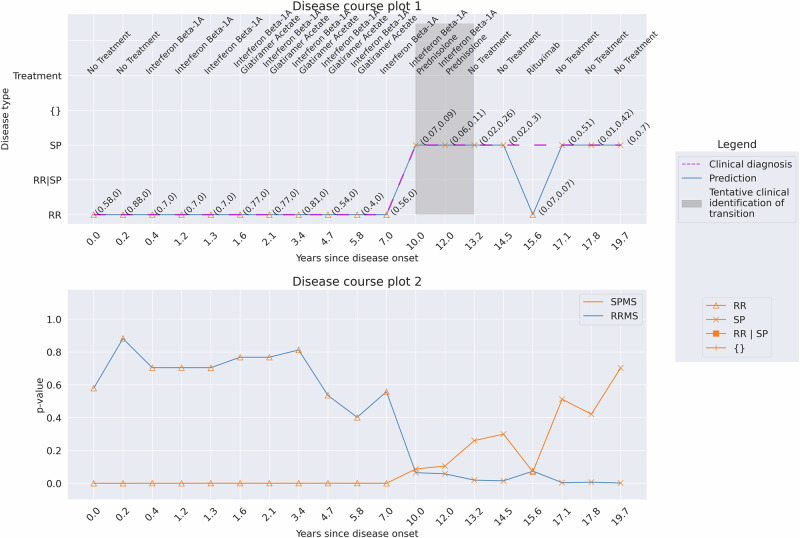
Patient 1. The prediction results at a confidence of 93% for a patient with 19 hospital visits (19.7 years). At four consecutive hospital visits, the patient was predicted SPMS with more than three months between the visits (years 10.0 and 14.5), followed by an RRMS prediction at year 15.6. This alternation is also associated with low p-values for RRMS and SPMS for the visits (disease course plot 2). The clinical identification of the transition occurred between years 10.0 and 13.2 (gray zone), whereas the model predicts the transition to be at year 10.0, aligning with clinical retrospective analysis.

**Fig. 9:**
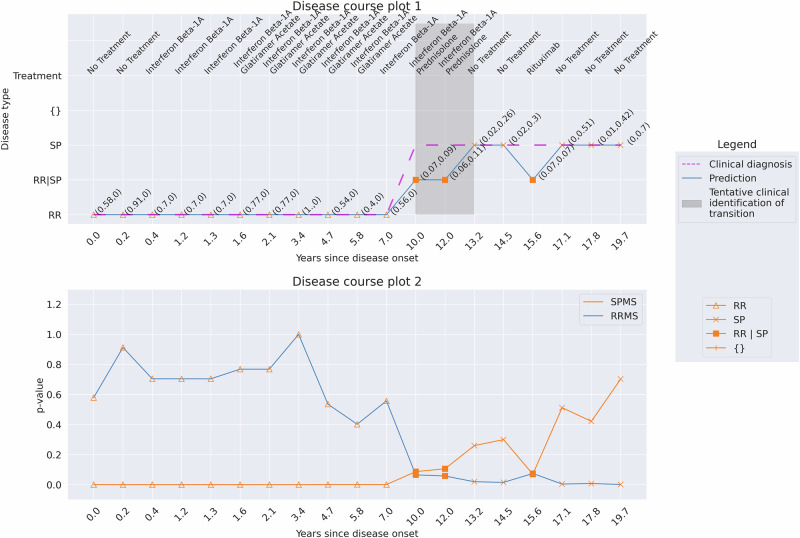
Patient 1. The prediction results at a confidence of 95% for a patient with 19 hospital visits (19.7 years). Increasing the confidence level from 93% to 95% allows for more multiple-label predictions between years 10.0 and 15.6. From year 17.1 and onwards, the model predicts only single-label SPMS. Here, the model does not make any errors but rather flags these visits 10.0, 12.0, and 15.6 for human analysis.

Since the evaluations made of the conformal predictor were made at a confidence of 93%, where the model’s single-label predictions were highest, an alternating prediction can thus indicate that the conformal predictor cannot assign correct single-label predictions for these cases (Fig. 8). To investigate this further, we analyzed these cases with 95% and 99% confidence, respectively. Increasing the confidence level led to an increase in the number of hospital visits where the patient was predicted to have multiple-label (RRMS | SPMS) (Fig. 9 and Supplementary Fig. 13). By predicting multiple-label means, the model makes no errors compared to clinical diagnosis. Similar observations were found for the remaining 22 cases (examples: Supplementary Figs. 14–28).

The results of the conformal predictor for predicting diagnosis at the hospital visits when in general, increasing the confidence from 93% to 95% and 99% are found in Table 3. There are only 382 single-label misclassifications at 99% confidence (error of 1.3%), compared to 2,073 at 93% (error of 7.3%), but multiple-label predictions (RRMS | SPMS) increased from 150 to 5001.

**Table 3:** Prediction on all the hospital visits on the test dataset with a confidence of 93%, 95%, and 99% compared to the clinical diagnosis

Predictions	93% confidence		95% confidence		99% confidence	
	RRMS (nv = 18,525)	SPMS (nv = 9798)	RRMS (nv = 18,525)	SPMS (nv = 9798)	RRMS (nv = 18,525)	SPMS (nv = 9798)
RRMS	19,616 (91.7%)	401 (5.8%)	19,351 (90.5%)	269 (3.9%)	18,371 (85.9%)	67 (1%)
SPMS	1672 (7.8%)	6484 (93.5%)	1168 (5.5%)	6116 (88.2%)	315 (1.5%)	4569 (65.9%)
Empty-{}	0 (0%)	0 (0%)	0 (0%)	0 (0%)	0 (0%)	0 (0%)
Multiple-label (RRMS | SPMS)	100 (0.5%)	50 (0.7%)	869 (4.1%)	550 (7.9%)	2702 (12.6%)	2299 (33.2%)

The predictions of RRMS and SPMS indicate single-label prediction, empty represents no valid prediction, and multiple-label represents both RRMS and SPMS prediction for the hospital visit. nv = number of hospital visits. *Percentages would not add up due to rounding off.

Since multiple-label predictions are when the model cannot distinguish between RRMS and SPMS to assign a single-label, these predictions indicate the patient is closer to or can be in transition to SPMS. We also found that 50 transitioning patients display non-typical disease progression, with a period of four to eight and even up to 17 years with multiple predictions, resembling an extended transition period for these cases. At a confidence level of 93%, 50 (1.4%) of all MS patients display this “atypical” disease progression, where changing the confidence level can be used to give feedback to a physician as a tool to aid in clinical decision-making.

### Publicly available web service

The model we have developed is based on retrospective data collected in Sweden. To aid in enabling our model to be available to other researchers, we have built a publicly available model with an interface named ‘MSP-tracker (multiple sclerosis progression-tracker)’. First, there was no statistically significant difference between the performance of the *model without MSIS* model and a model that only included basic (Supplementary Table 1, Basic info) and relapse-related information (Supplementary Table 1, relapse data), ‘*Basic Info+Relapse*’ (Supplementary Figs. 3 and 4). Secondly, we removed all possibly identifiable information from the data; the year of birth was used instead of the exact date, and all other information with dates was reduced to year and month, making an anonymized model. We found no significant loss in performance difference between the anonymized *MSP-tracker* and its counterpart ‘Basic Info + Relapse’ (*p*-value = 0.89), indicating no decay in performance by anonymizing the data.

The anonymized version of the model is available online (https://msp-tracker.serve.scilifelab.se/) and configured to accept up to 25 hospital visits. The web server can receive either direct input or from uploaded CSV files. The model can also be used at user-defined confidence levels, with output results displayed as disease course plots. The model explanation using SHAP is available post-prediction, yielding both global interpretations of the input data and individual interpretations at each hospital visit.

## Discussion

Having a clear understanding of the disease course and its current state is essential in MS, as available treatments and treatment goals vary depending on the phase of the disease. Though there are many disease-modifying treatments for RRMS, the treatments used for SPMS are few, with relatively limited efficacy^25,26^. The identification of the transition from RRMS to SPMS is made retrospectively, often with a delay of several years, and still remains a challenge^1^. Therefore, early recognition of patients with a risk of progressive disease could enable timely, meaningful interventions and also restrict unnecessary exposure to medications with associated side effects in the longer term.

**Fig. 10:**
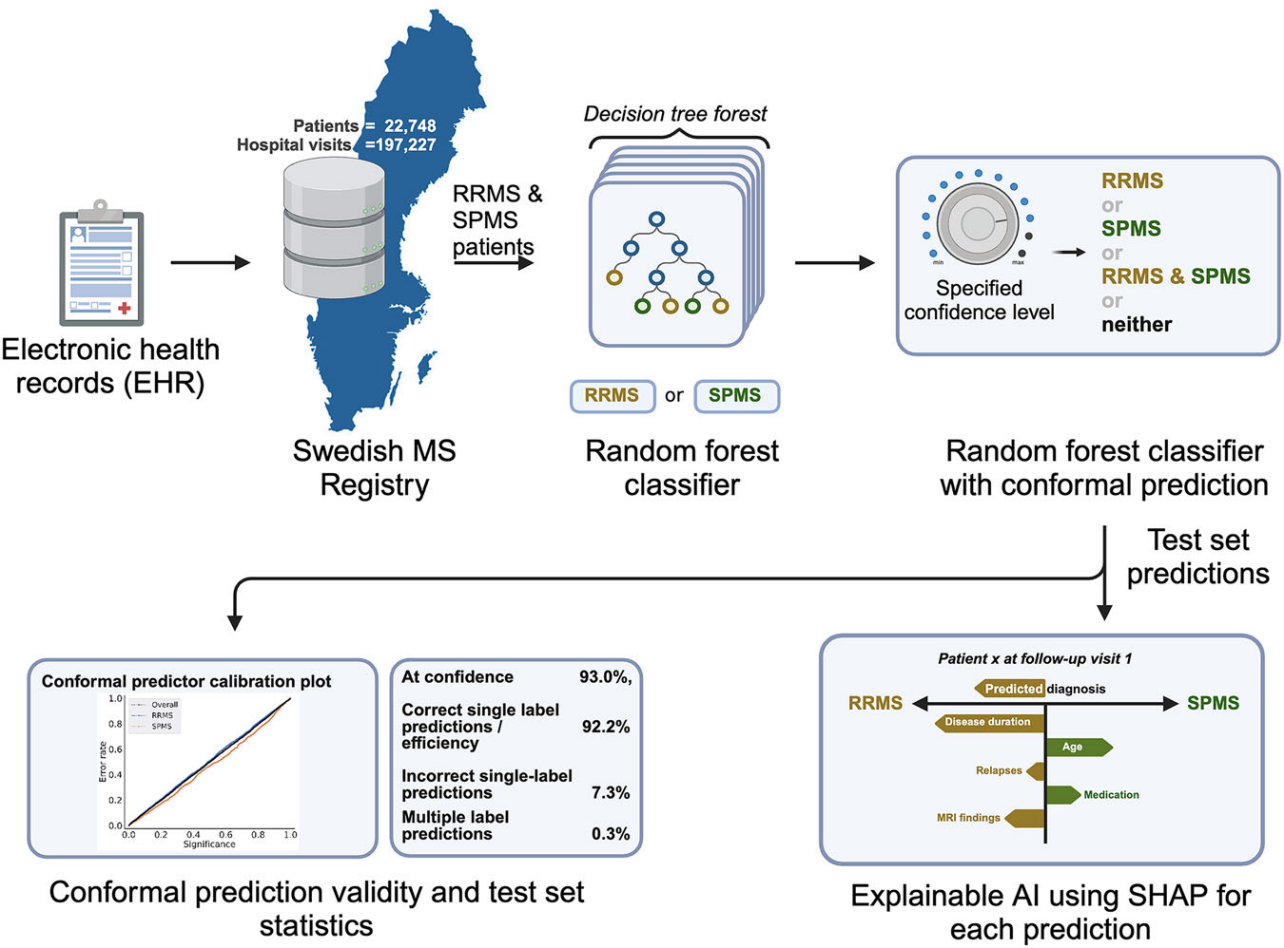
Summary figure illustrating a comprehensive view of the entire study. A predictive model capable of distinguishing between RRMS and SPMS was trained on EHR data collected during hospital visits in Sweden. CP was incorporated to provide valid measures of uncertainty for predictions at the individual patient level. The model showed proof of validity and exhibited high performance on the test set data. Additionally, SHAP was utilized to understand the contribution of features in each prediction. Created with BioRender.com. Kultima, K. (2024) https://BioRender.com/s87n810.

We present here a first-of-its-kind predictive model that is able to distinguish between RRMS and SPMS at high accuracy, trained on data from EHR collected at routine hospital visits. To enable future usefulness in clinical settings and research, we applied CP to deliver valid measures of uncertainty in predictions on individual patient levels. We successfully produced a theoretical and empirically valid model with the highest efficiency at a 93% confidence level and demonstrated on an external test set that it enables effective prediction of a patient’s clinical course with individual confidence measures (Fig. 10).

Clinical AI tools must convey predictive uncertainty for each individual patient^17^. We have recently demonstrated that CP-enabled AI can support predictions with user-defined confidence^20^. A well-performing CP should ideally generate fewer unreliable predictions. In this study, the model delivers 81% single-label predictions and 18% unreliable predictions at 99% confidence, marking the error rate around 1%. When faced with unreliable predictions, the explainable AI becomes essential to elucidate the reason behind these predictions. Both CP and explainable AI equip tools for users to scrutinize and analyze the occasionally unreliable predictions. Here, unreliable predictions are identified, thus allowing deeper analysis by an expert neurologist.

The clinical course for the transition from RRMS to SPMS has been defined by Lublin et al.^27^ as the “progressive accumulation of disability after [an] initial relapsing course” assessed, at a minimum, annually^28^. As no “gold standard” criteria exist beyond this^27,29^, the SPMS diagnosis ultimately rests on the individual clinician’s judgment, primarily using the patient’s history and the clinical examination. Lorscheider et al.^30^ have since further developed the definition of SPMS by assessing the performance of standardized criteria against the “ground truth” of a consensus diagnosis by independent, expert neurologists. Multiple permutations of seven different EDSS-related criteria were generated and tested against the independent, consensus diagnosis with the best-performing permutation proposed as standardized criteria for SPMS, demonstrating performance comparable to the physician’s diagnosis.

While not widely used in clinical practice, their standardized criteria did show a notably higher sensitivity than the physician diagnosis and identified SPMS patients around three years earlier than the physician, though the specificity was lower. Using these criteria, it has been demonstrated that at the time of SPMS diagnosis, individuals will typically have EDSS scores above 4^29,30^ with disease durations greater than ten years^30^. The importance of the EDSS score in determining the transition from RRMS to SPMS is also reflected in our model, which had the largest contribution to predictions. On the other hand, other factors, such as the age at visit or time since diagnosis, also contributed substantially to the predictions in our model, but were not considered as part of the criteria described by Lorscheider et al. More importantly, the now proposed approach enables objective predictions, trained, validated, and calibrated on more than 10,000 patients with over 85,000 hospital visits. Each prediction is complemented with a measure of uncertainty, and the CP enables tracking the disease progression over time.

Multiple approaches have been described to predict the conversion from RRMS to SPMS^10,14,15,31^. In the study by Manouchehrinia et al., data from multiple cohorts was utilized, and the prediction method was specifically designed to predict the risk of conversion for patients with RRMS^15^. This model showcased an accuracy of 77–87% to predict the risk of conversion to SPMS in 10, 15, and 20 years. In a similar study by Seccia et al., which predicted the transition of RRMS patients within 180, 360, and 720 days, the model reached a maximum recall of 100% and a precision of 9%. However, the model may not be applicable to patients initially diagnosed with SPMS, a limitation shared with studies by Manouchehrinia et al. and Skoog et al.^10^. As such, these studies differ from ours since they try to predict the future risk of transition, rather than categorizing patients based on current data. However, the study by Ziemssen et al. addresses the above limitation by categorizing patients as RRMS, SPMS, or transitioning using EHR and questionnaire data^31^. Their study on 198 individuals achieved a sensitivity of 82% and a specificity of 84% in identifying transitioning patients. In comparison, our study utilized 14,382 individuals and exhibited results with a substantially higher sensitivity and specificity of 93%. Moreover, we implemented an uncertainty measure for each hospital visit of a patient. Importantly, our model does not rely on additional questionnaire data and was able to demonstrate the progression of the disease for individual patients, which sets it apart from previous approaches. Moreover, the other models are susceptible to systematic differences between the training and external data or data drift over time. Using CP provides a robust means of handling uncertainties and addressing potential shifts in data over time^20^. However, it is crucial to examine the model’s performance in detail to identify any potential weaknesses and areas for improvement.

We conducted a deep analysis of the frequently misclassified patients, which revealed key insights into the strengths and limitations of our model in comparison with the registry data. In the majority of misclassified RRMS patients, the model classified the patient as transitioning from RRMS to SPMS earlier than according to the registry data. In these cases, these ‘misclassifications’ may instead represent early prediction by the model or a diagnostic delay in the registry data. Conversely, it struggled with certain patient profiles, particularly in patients with higher EDSS post-relapse. Additionally, for most of the misclassified SPMS patients, the transition to SPMS was found earlier in the registry data than predicted by the model. This was particularly observed in young adults and/or those with lower EDSS scores, which are relatively uncommon characteristics in SPMS. This discrepancy highlights the importance of considering the clinical context alongside the model’s predictions, particularly in less typical scenarios.

CP proves valuable in recognizing new data that deviates from the characteristics of the training data. This is relevant when predicting an external dataset or when encountering data that the model has not seen before. This study has trained the model on registry data from over 850 clinicians and more than 60 Swedish hospitals. Although the model exhibits notable performance, this might not hold true when predicting external data or on unfamiliar cases within Sweden, signifying the use of CP to identify these. More than 80% of all Swedish MS patients are present in the Swedish MS registry, and data from other countries has not been part of this study, so generalization at large poses a challenge that needs to be addressed. Data drift over the years can also lead to an increase in unreliable predictions, which warns the recalibration necessary for the underlying AI and CP system.

The major strength of this study is using CP for predicting disease transition and disease state at each visit, thereby outlining the disease course of a patient. By basing predictions on clinical data already collected during hospital visits, the need for additional data collection, such as biological markers or questionnaires, is eliminated, thereby facilitating easier implementation and integration of the model in healthcare and research settings. Moreover, the model was also integrated with explainable AI, facilitating easier interpretation and assignment of labels for predictions regarded as unreliable.

The limitation of this study is the absence of analysis of the prospective data collected from the clinics. By conducting prospective data analysis, the practicality of the model at the clinics can be evaluated. Moreover, the model has not undergone evaluation using external data from other cohorts outside of Sweden. Validating CP on external data could show the potential of the model. To aid in this process, an anonymized version of the model is available online (https://msp-tracker.serve.scilifelab.se/).

## Methods

### Ethical approval

This study was conducted in accordance with the ethical principles outlined in the Declaration of Helsinki (WMA, 2024). This study was approved by the Swedish Ethical Review Authority (Dnr 2021-00702). The data used was anonymized registry data and the informed consent was waived as participants had already provided written consent for their data to be used for research purposes.

### Dataset and quality control

The data was obtained from SMSreg^23^, containing 22,748 patients with 197,227 hospital visits, with clinical measurements collected during each hospital visit. The data was cleaned for duplicates and missing essential data points for expanded disability status scale (EDSS) score, date of birth, progress during each visit such as RRMS/SPMS/PPMS, and debut date. The data comprises patients with transition (initial diagnosis as RRMS and final diagnosis as SPMS), RRMS (initial and final diagnosis as RRMS), and SPMS (initial and final diagnosis as SPMS). For the RRMS patients, all the visits within two years of the last visit were removed as their clinical endpoint had not yet been determined. This ensures the removal of all the unidentified transitions from the data. After quality control and removal of PPMS patients, 17,045 patients with 143,053 hospital visits were retained.

A hospital visit consisted of age and EDSS measured at the visit. For each visit, the last collected clinical measures such as treatment, clinical assessment tests, relapse data, MRI data, and MSIS data (Supplementary Table 1) were appended, along with the age at which these measures were collected. For therapeutics, the drugs/treatments were categorized into first-line, second-line DMT, relapse treatment drugs, stem cell treatment, and any other drugs (Supplementary Table 1).

For data relating to relapses, the total number of occurrences of different categories of relapses (including unilateral optic neuritis, sensory/afferent monofocal relapse, multifocal relapse, and relapses requiring steroid treatment) were summed up until the day of the hospital visit before appending. Additional information regarding treatment received for the relapse and remission of the last relapse was also included as binary variables. For the MRI data, the number of T2-weighted lesions and the number and site of T1-weighted gadolinium-enhancing lesions (i.e., brain vs. spinal cord) were considered. Each type of lesion was binned into three groups based on the number of lesions present at the hospital visit: (1) ≤9, (2) >9, and ≤20, (3) >20 lesions.

For a patient, during the initial hospital visit, EDSS, age at visit, age at diagnosis, age at debut relapse, and sex were recorded. However, certain parameters may be missing as they have not yet been measured. The missing values, including those for other parameters of the patient, were imputed using the value −1.

### Data splitting

There were three possible types of data for a patient: a patient having hospital visits with only RRMS, only SPMS, or RRMS at debut, and SPMS at the latest. To maintain an even distribution of these patients across the data splits, a stratified split was applied, grouping all the visits associated with a patient in the same split of the data. Thereby dividing the data into four subsets: training (65%), validation (5%), calibration (5%), and test (25%) datasets (Table 4). The validation set is created to optimize the deep learning model, and therefore, for traditional machine learning models, the validation dataset is merged with the training set for training. For cross-validation used in this study, the training and validation sets are merged for both deep learning and machine learning models and used for training.

**Table 4:** Data splits created from SMSREG data

Dataset	Number of patients	Number of hospital visits	Number of RRMS patients	Number of SPMS patients	Number of transitioning patients
**Training set (65%)**	9348	74,064	6414 (69%)	1696 (18%)	1238 (13%)
**Validation set (5%)**	719	5626	491 (68%)	125 (17%)	103 (14%)
**Calibration set (5%)**	720	5771	467 (65%)	152 (21%)	101 (14%)
**Test set (25%)**	3595	28,323	2451 (68%)	677 (19%)	467 (13%)

The dataset is divided into train, valid, calibration, and test sets, each containing unique patients and their EHR.

### Architectures

Five model architectures were utilized for predictions: (1) logistic regression (LR), (2) support vector machines (SVM), (3) gradient-boosting (GB), (4) random forest (RF), and (5) a DL model using long short-term memory (LSTM). For SVM, GB, and RF, grid-search CV was conducted to identify the best-performing parameters for the models. For SVM, the radial basis function (RBF) kernel was employed, with the parameter gamma set to 0.0001 and the regularization parameter C set to one. In the case of GB, the number of estimators was 50, a minimum sample split of two with the criterion set to “friedman_mse” with exponential loss. For the RF architecture, the minimum number of samples per leaf was five, the criterion was Gini with an ensemble of 150 estimators.

The deep learning model was a hybrid of an LSTM network and a multi-layer perceptron. The LSTM consisted of a single layer with a hidden cell size of 256, processing values related to specific hospital visits. The multi-layer perceptron comprised two layers of eight neurons, which were used to handle patient-related, visit-independent values, such as sex label, diagnosis age, and age at first relapse. The output from LSTM and multi-layer perceptron were concatenated and further processed using a two-layered perceptron with 128 neurons and an output size of 2. ReLU served as the activation function, and the model utilized cross-entropy loss and Adam optimizer with a learning rate of 0.0001.

### Conformal prediction

Conformal prediction (CP) is a framework built on top of any ML model to retain the error rate of the prediction to a pre-specified level^19^. CP is model agnostic (meaning, it can be implemented on all models) and is implemented on top of a prediction algorithm. Unlike single-valued output from a prediction algorithm, CP produces a prediction region containing a set of class labels for classification and a confidence interval for regression. Using CP, a non-conformity measure $\alpha$_i_ is calculated for an object *i* using a non-conformity function *h(x*_*i*_*)*, where *x* represents the features and *h* represents a scoring algorithm such as a machine learning algorithm. When applied to a classification problem, at first, non-conformity $\alpha$_i_ is calculated for all the n instances in the calibration set, yielding $\alpha$_1_, …,$\,\alpha$_n_. During the prediction phase, the non-conformity$\quad \alpha$_n+1_ from a test instance is used to calculate a set of p-values for each class label using Eq. 1, which ranks the$\quad \alpha$_n+1_ against all the $\alpha$_1_, …,$\,\alpha$_n_. Using a statistical test and employing a confidence cutoff (1-significance), such as 95%, implying a significance of 0.05, all the labels with a *p*-value greater than or equal to 0.05 are included in the output prediction, resulting in single-label, multiple-label, or empty predictions.

1 $${p}_{n+1}=\frac{|j=1,...,n+1:{\alpha }_{j}\ge {\alpha }_{n+1}|}{n+1}$$

A CP p-value differs from a traditional *p*-value. Typically, the traditional *p*-value assesses the evidence against a null hypothesis. In contrast, CP *p*-values measure the non-conformity of a new observation to a given calibration dataset. Specifically, the CP *p*-values indicate the proportion of calibration examples that exhibit greater non-conformity than the new observation when assigned a particular label. A low CP *p*-value suggests that the new observation is unusual compared to calibration data for that particular label. Essentially, CP *p*-values provide insights into how well a new observation fits within the existing data, rather than directly addressing the clinical or research question at hand.

For a binary classification, the possible output from CP are {0}, {1}, {0,1}, or {}. A smaller prediction region with only a single-label ({0} and {1}) is more desired and efficient, for explaining the output of the model. A multiple-label prediction ({0,1}) is when multiple class labels have higher confidence, and the model is unable to determine between the two class labels. This occurs when uncertainty arises in assigning a single-label for the prediction. Although these predictions can be harder to interpret, they are not incorrect per se. These can be interpreted as unreliable/uncertain predictions and can be flagged for manual or expert inspection to determine the correct class label. The empty set ({}) predictions are obtained if the confidence is low on both class labels, and it occurs when the input data differs from the data the model is trained on. This can highlight systematic differences between training and external data or data drifts that happened over time.

The desired CP confidence can be set by the user during the prediction time. At higher confidence, the probability of having the correct label in the output prediction set increases, yielding a wider prediction region (increase in multiple-label predictions). Likewise, lower confidence produces a smaller prediction region (increase in single-label predictions and empty predictions).

There are two ways of calibrating a conformal predictor: (1) transductive framework and (2) inductive framework. In transductive CP, for each new instance during the predictive phase, all the data is used to calculate the conformity score, making it necessary to retrain the model for every data point in the calibration and test set. Though this method is more robust to outliers and anomalies in the dataset, the computational demand makes it unusable for large datasets and deep learning algorithms. Inductive CP (ICP), on the other hand, is built using training and calibration datasets and is applied to the test dataset. The calibration dataset is identically independently distributed (IID) data from the training dataset. The lower computational demand and easiness of recalibrating the model make ICP popular in many fields.

In this study, we use the ICP framework, by using 5% of the available data as a calibration dataset. The basic implementation of CP considers the error rate on a population level. Making the error rate on one label to be lower than the other label. To overcome this, Mondrian CP was used to achieve a predefined error rate within each class label. Instead of tuning on the entire population, the CP was tuned on each class label. This enables reliability in prediction on an individual level, making the model applicable for clinical use, as we are more interested in individual predictions than population-level prediction in a clinical setting.

### SHapley Additive exPlanations

SHapley Additive exPlanations (SHAP) uses a game theoretic approach to generate explainable and interpretable output from a machine learning model^22^. Using this framework, SHAP values can be calculated for each feature in a data point by contrasting predictions with and without the presence of a specific feature. This process is iteratively applied for the entire dataset, resulting in the generation of SHAP values for each feature across the dataset. The difference in the impact of the feature for a prediction reveals positive or negative contributions for both individual predictions and the prediction on the entire dataset. Thus, SHAP allows us to calculate both the global interpretation, giving insight into the overall importance of features in the dataset, and also for the individual predictions, interpreting the rationale behind the output using feature contribution.

Providing explanations for individual predictions holds substantial importance within clinical settings. This offers a better understanding and increases the reliability of the predictions^32^. In this study, we explain each prediction using force plots. In these plots, SHAP values for individual features are plotted along the *x*-axis, where each feature is represented by a bar, with the length of the bar corresponding to the magnitude of the feature’s impact and the colors indicating positive values in red (SPMS) and negative values in blue (RRMS). The visual nature of these plots helps to spot the most influential features driving the predictions and also to get a sense of how features interact to influence the outcome.

In contrast, the global understanding of the model is achieved using a summary plot and a beeswarm plot. Both these plots provide a comprehensive overview of the importance of features in the entire dataset. The *y*-axis displays features ranked according to their importance, with features having a higher impact on the predictions at the top. The *x*-axis in the summary plot represents mean absolute SHAP values, displaying the global importance of the features. In the beeswarm plot, the *x*-axis represents the SHAP values and their importance, color-coded according to feature value. The SHAP value of a feature from each data point is plotted, with overlapping SHAP values jittered in the *y*-axis to accommodate and form a distribution.

## Supplementary information

Supplementary

## Data Availability

The data used in the study cannot be shared to protect the privacy of the individuals. All the data can be obtained by applying through the Swedish MS Registry (https://neuroreg.se/forskning/datauttag-for-forskningsandamal/).

## Code availability

The code used for data pre-processing, the final model, and the web server are available at https://github.com/caramba-uu/MSP-tracker.git.
