# Supplementary material for: Conformal prediction enables disease course prediction and allows individualized diagnostic uncertainty in multiple sclerosis
Source: NPJ Digit Med. 2025 Apr 24;8:224. doi: 10.1038/s41746-025-01616-z (PMC12022056; doi:10.1038/s41746-025-01616-z)
Supplement: Supplementary file 1 — Supplementary [file 41746_2025_1616_MOESM1_ESM.docx]

Supplementary Information

**Supplementary Table 1: All the derived features from the clinical features used for training of the model.** The data contained missing values for clinical assessment tests (60-73%), relapse data (12%), MRI data (26%), and MSIS-29 (59%). These missing values were imputed to form the complete dataset.

| **Feature** | **Feature type** |
| --- | --- |
| **Visit information (Basic info)** | |
| **Diagnosis age** | Approximate age at which the disease was diagnosed |
| **EDSS score** | EDSS score measured during the visit |
| **Age at visit** | Age of the patient at the visit when the EDSS score was given |
| **Patient information** | |
| **Sex label** | Sex of the patient |
| **Drug treatment** | |
| **No treatment** | Whether the patient has not received any treatment during this visit |
| **First-line DMT** | Whether the patient is undergoing first line (disease-modifying therapies) DMT |
| **Second-line DMT** | Whether the patient is undergoing second-line DMT |
| **Other drugs** | Whether the patient is consuming any other drugs/treatment |
| **Relapse treatment drugs** | Whether the patient is receiving drugs for relapse |
| **Stem cell treatment** | Whether the patient has received hematopoietic stem cell transplantation |
| **Clinical assessment tests** | |
| **EQ5D score** | EQ5D score during the visit |
| **Age at EQ5D** | Age at EQ5D was given |
| **SDMT score** | Symbol Digit Modalities Test (SDMT) score during the visit |
| **Age at SDMT** | Age at SDMT was given |
| **Relapse data** | |
| **Mono on sum** | Sum of unilateral optic neuritis relapses |
| **Monofocal sum** | Sum of other monofocal relapse |
| **Multi focal sum** | Sum of multifocal relapse |
| **Afferent non on sum** | Sum of sensory/afferent non-optic neuritis relapses |
| **Steroid treatment sum** | Sum of steroid treatments received |
| **Is last relapse steroid treated** | If the last relapse was treated with steroids |
| **Is last relapse completely remitted** | Was the last relapse completely remitted |
| **Age at debut relapse** | Age at first relapse |
| **Age at relapse** | Age at relapse |
| **MRI data** | |
| **T2 lesion category** | Number of T2-weighted MRI lesions (binned) |
| **Brain barrier lesion category** | Number of T1-weighted gadolinium-enhancing brain lesions (binned) |
| **Spinal barrier lesion category** | Number of T1-weighted gadolinium-enhancing spinal cord lesions (binned) |
| **Age at MRI** | Age at the time that the MRI was performed |
| **MSIS data** | |
| **MSIS 01 - MSIS 29 (29 questions)** | [Hobart](http://paperpile.com/b/5FC7BV/lgUJk) et al[^1^](https://paperpile.com/c/5FC7BV/lgUJk) |
| **MSIS physically** |  |
| **MSIS psychologically** |  |
| **MSIS physically 100** |  |
| **MSIS psychologically 100** |  |
| **Age at MSIS** | Age at the time that the MSIS was administered |


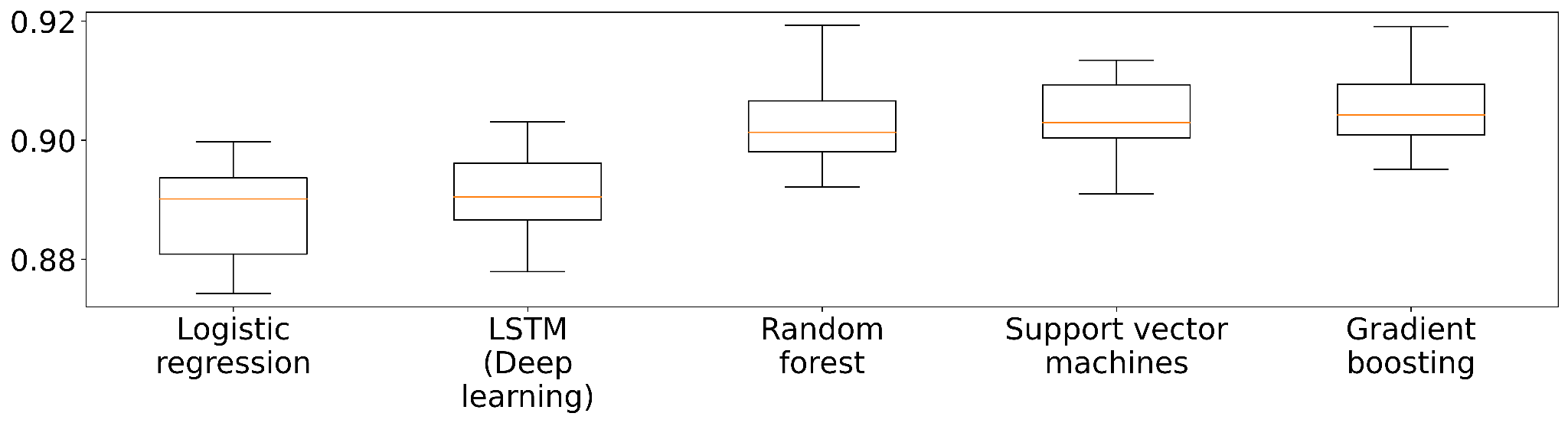


**Supplementary Figure 1:** **Box plot showing F1 scores from the 10-fold CV of traditional machine learning models and a deep learning model.** The random forest, support vector machines, and gradient boosting had better performance when compared to logistic regression and deep learning models. All the models showed an average F1 score of >0.88.


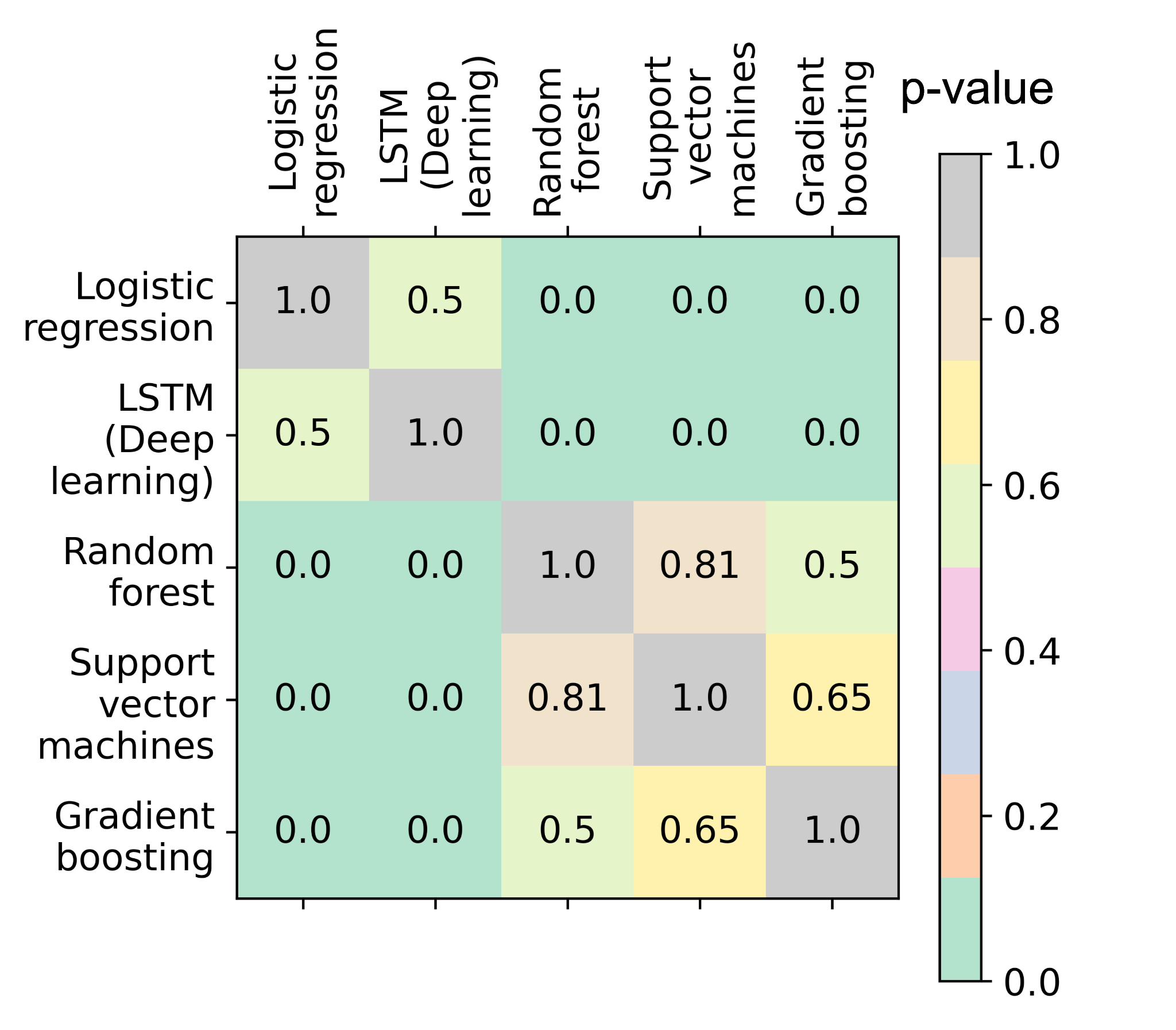


**Supplementary Figure 2: Randomization test (p-value<0.05) on F1 scores from the 10-fold CV of traditional machine learning models and a deep learning model.** Significant p-values represent a higher or lower performance between the models. Comparing the box plot Supplementary Figure 1, random forest, support vector machines, and gradient boosting models performed significantly better than both the deep learning model and logistic regression.


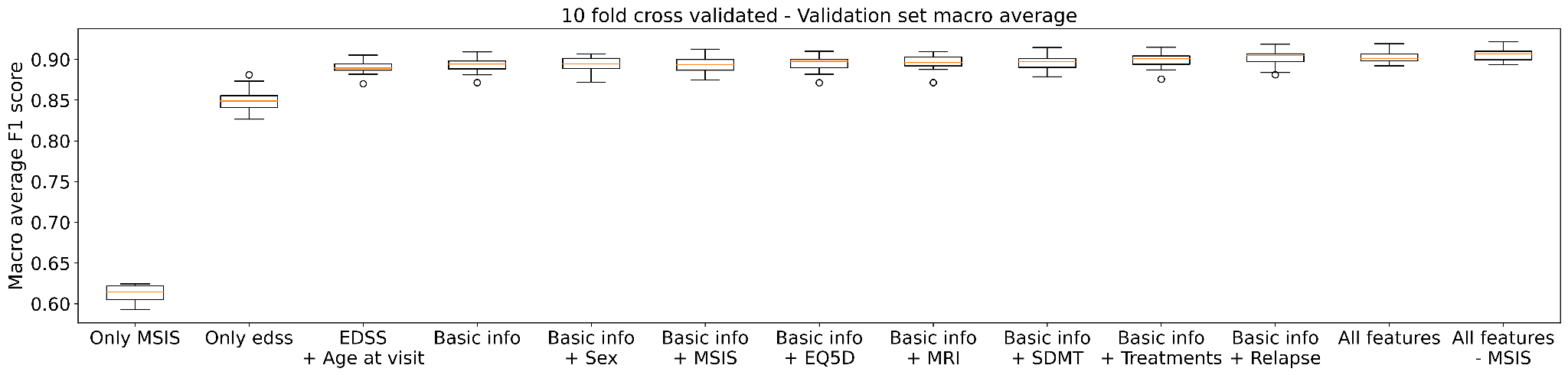


**Supplementary Figure 3: Box plot showing F1 scores from the 10-fold CV of RF models trained on a combination of features.** With EDSS alone, the model achieved a macro average F1 score of approximately 0.85. The model was evaluated with the inclusion of additional features.


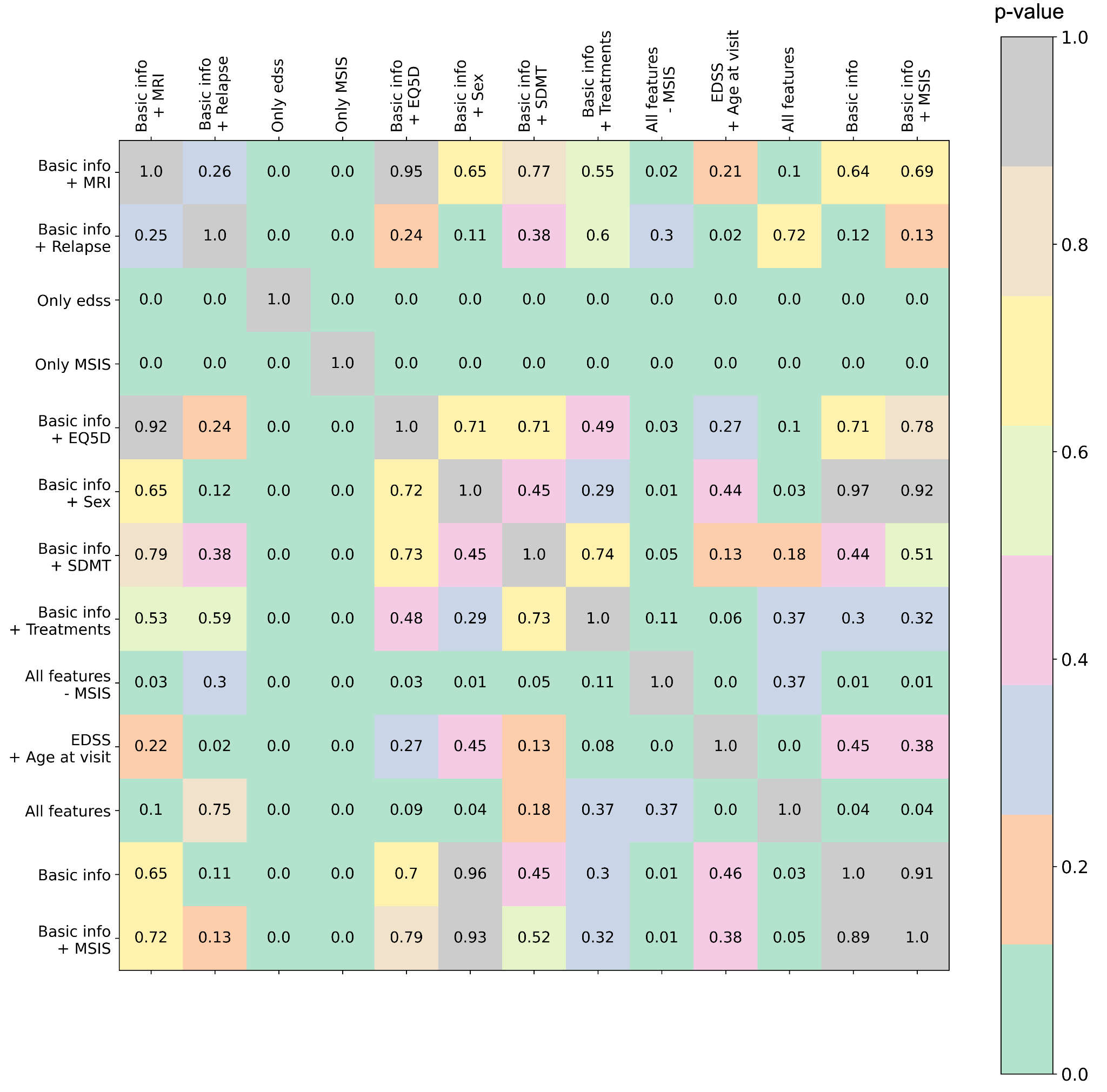


**Supplementary Figure 4: Randomization test (p-value<0.05) on F1 scores from the 10-fold CV of RF models trained on a combination of features.** Significant p-values represent a higher or lower performance between the models. Comparing Supplementary Figure 3, the features set “All features - MSIS” has a significantly better performance than all the other combinations except “Basic info + relapse”, “All features”, and "Basic info + Treatments”. For "Basic info + SDMT", although the figure shows p-value of 0.05, this is a result of rounding from the actual value of 0.049, which makes it significantly different from “All features - MSIS”.


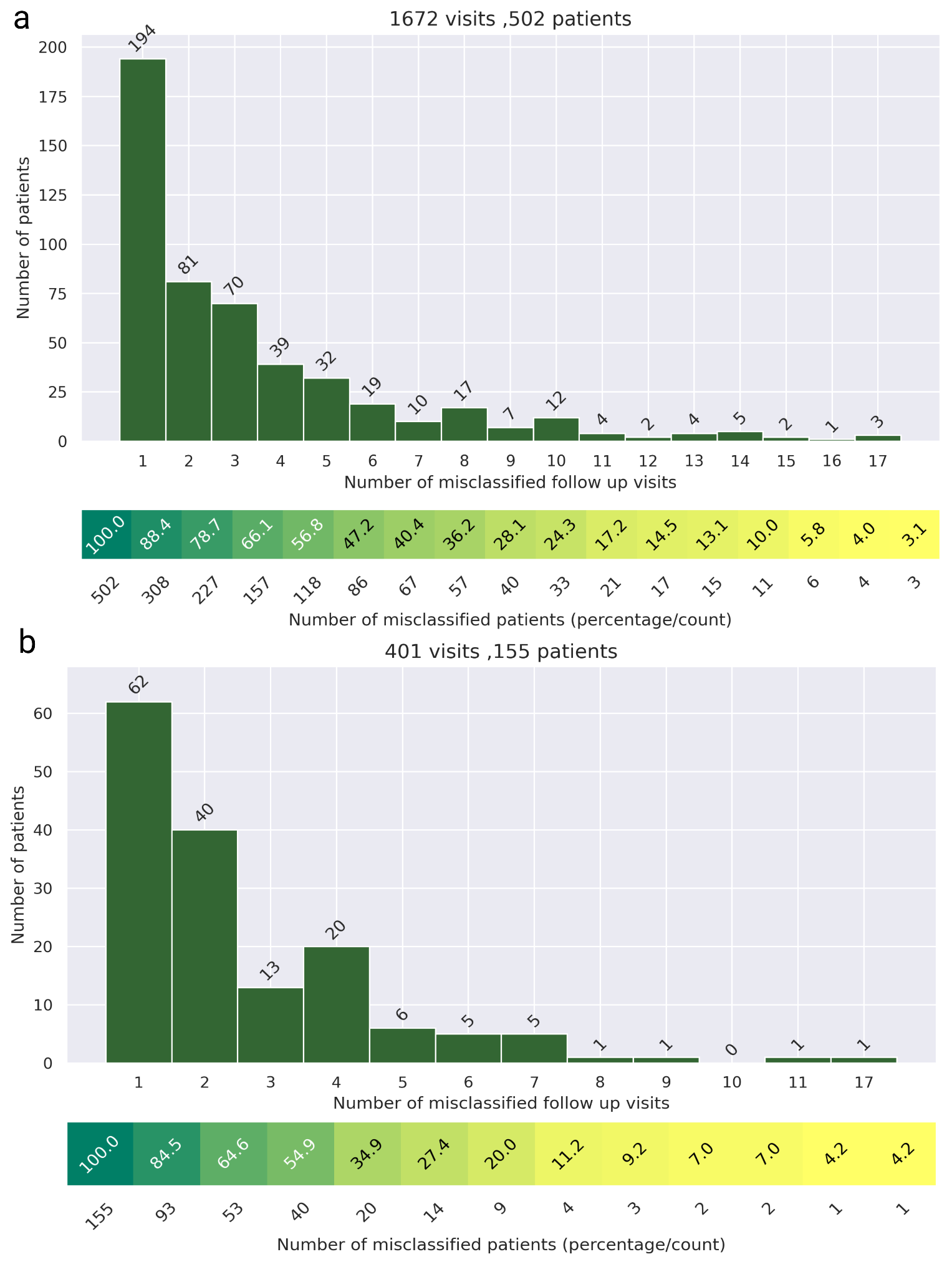


**Supplementary Figure 5:** **Patients and the number of misclassified hospital visits.** The x-axis represents the number of misclassified hospital visits, and the y-axis represents the patients in the test set. The cumulative contribution to the erroneous prediction by patients based on the number of misclassified visits is given below. (a) The misclassification of 1,672 hospital visits from 502 patients diagnosed with RRMS and predicted SPMS. 56.8% of misclassification is from 118 patients, and the rest of the errors are from 384 patients. (b) The misclassification of 401 hospital visits from 155 patients diagnosed with SPMS and predicted RRMS. 40 patients contribute to 54.9% of the misclassification, and the remaining 45.1% of errors come from 115 patients.


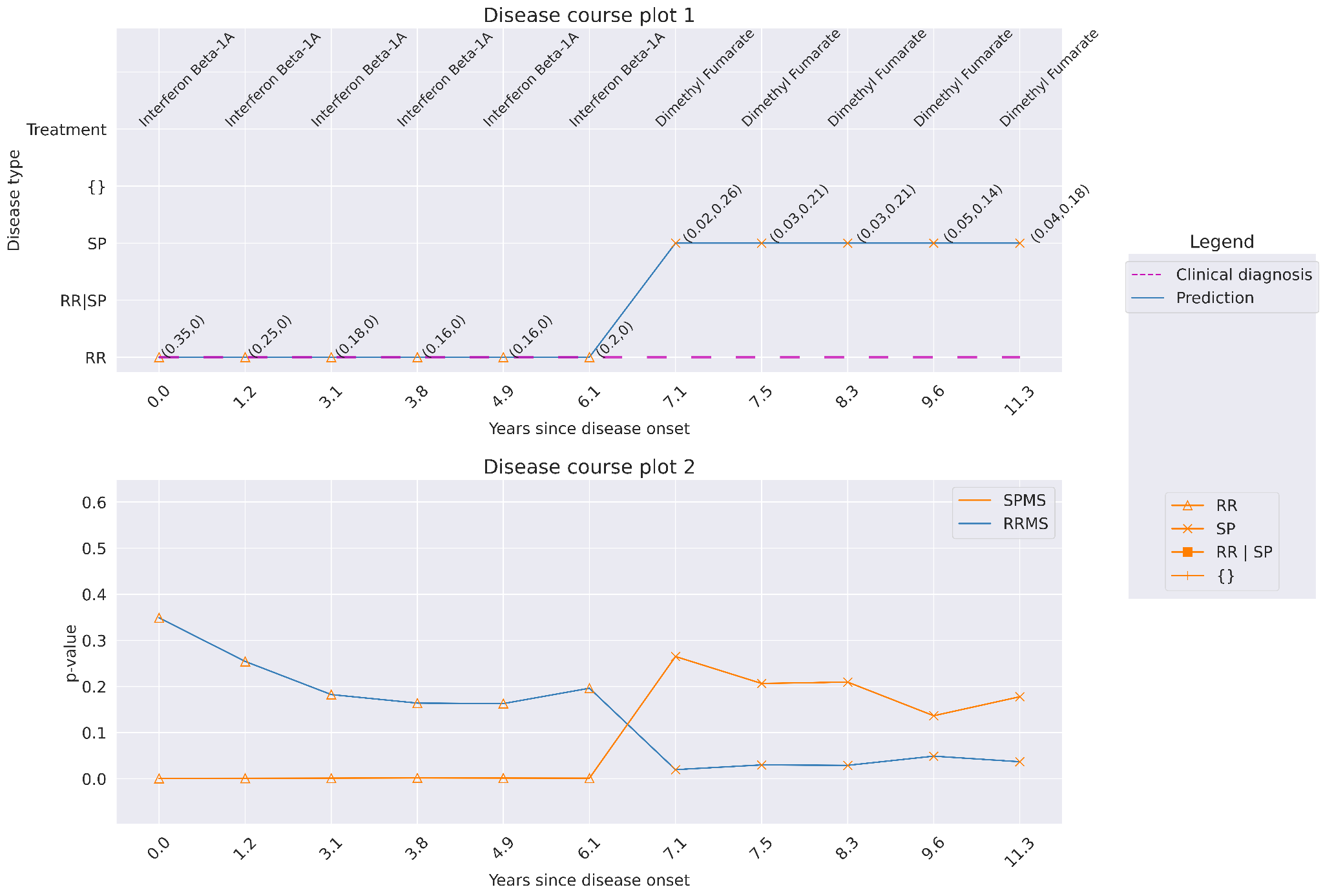


**Supplementary Figure 6: Predictions at a confidence of 93% for a patient with a disease course of 11.3 years over 11 hospital visits.** The patient was diagnosed with RRMS at the onset and at the latest hospital visit, while the prediction shows the patient has transitioned to SPMS at the hospital visit year 7.1 (Disease course plot 1). From Disease course plot 2, there is an apparent decrease in RRMS p-value between the hospital visit years 0 to 7.1, while there is an increase in SPMS p-value from the year 6.1 to the last hospital visit (11.3 years), suggesting a transition from RRMS to SPMS.


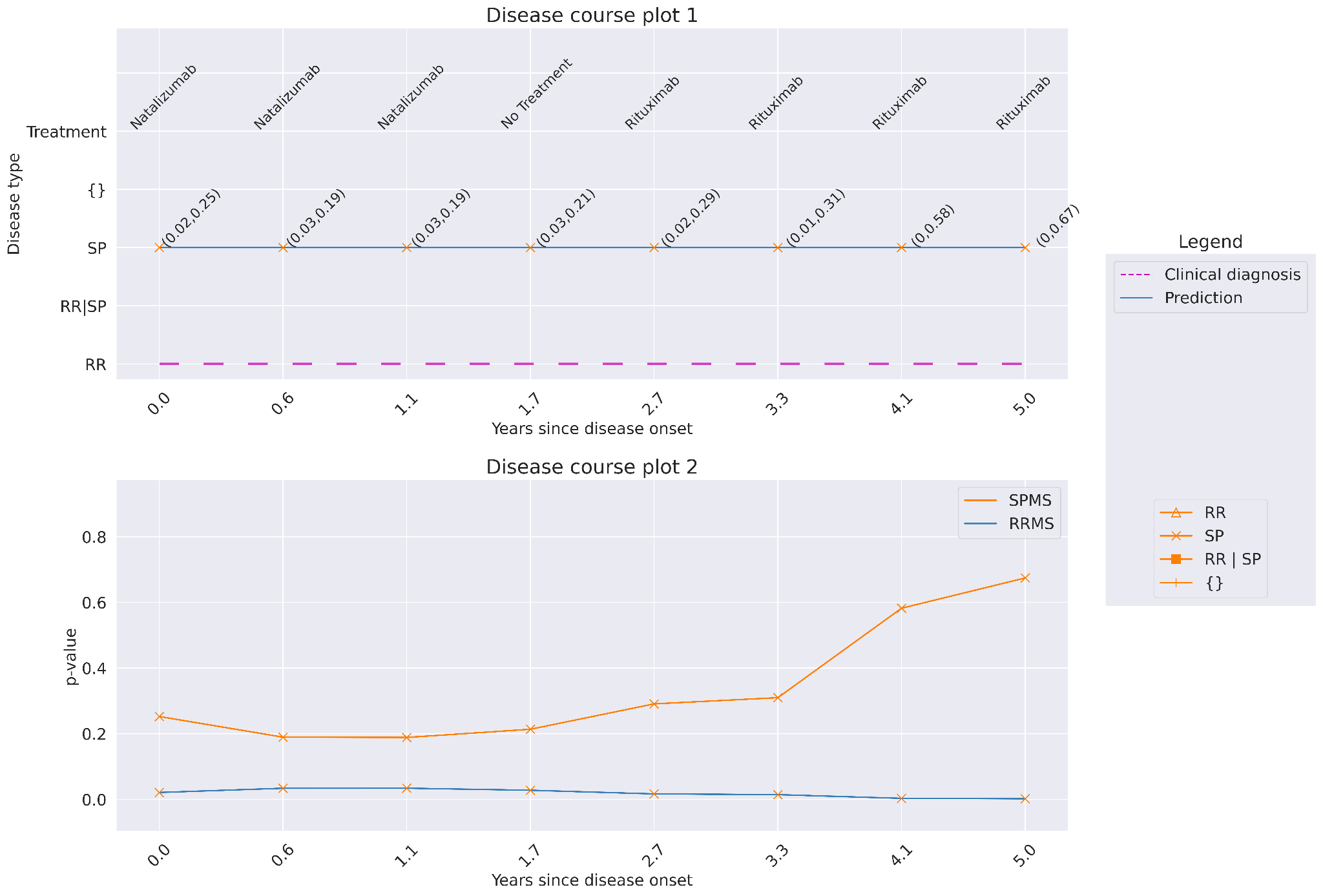


**Supplementary Figure 7: Predictions at a confidence of 93% for a patient with a disease course of 5 years over 8 hospital visits.** The patient was diagnosed with RRMS at the onset and at the latest hospital visit, while SPMS was predicted to be present from the onset (Disease course plot 1). From the Disease course plot 2, there appears to be an increased SPMS p-value compared to the RRMS p-value. Additionally, there is an increase in SPMS p-value from year 0 to year 5, suggesting the patient may have already transitioned to SPMS.


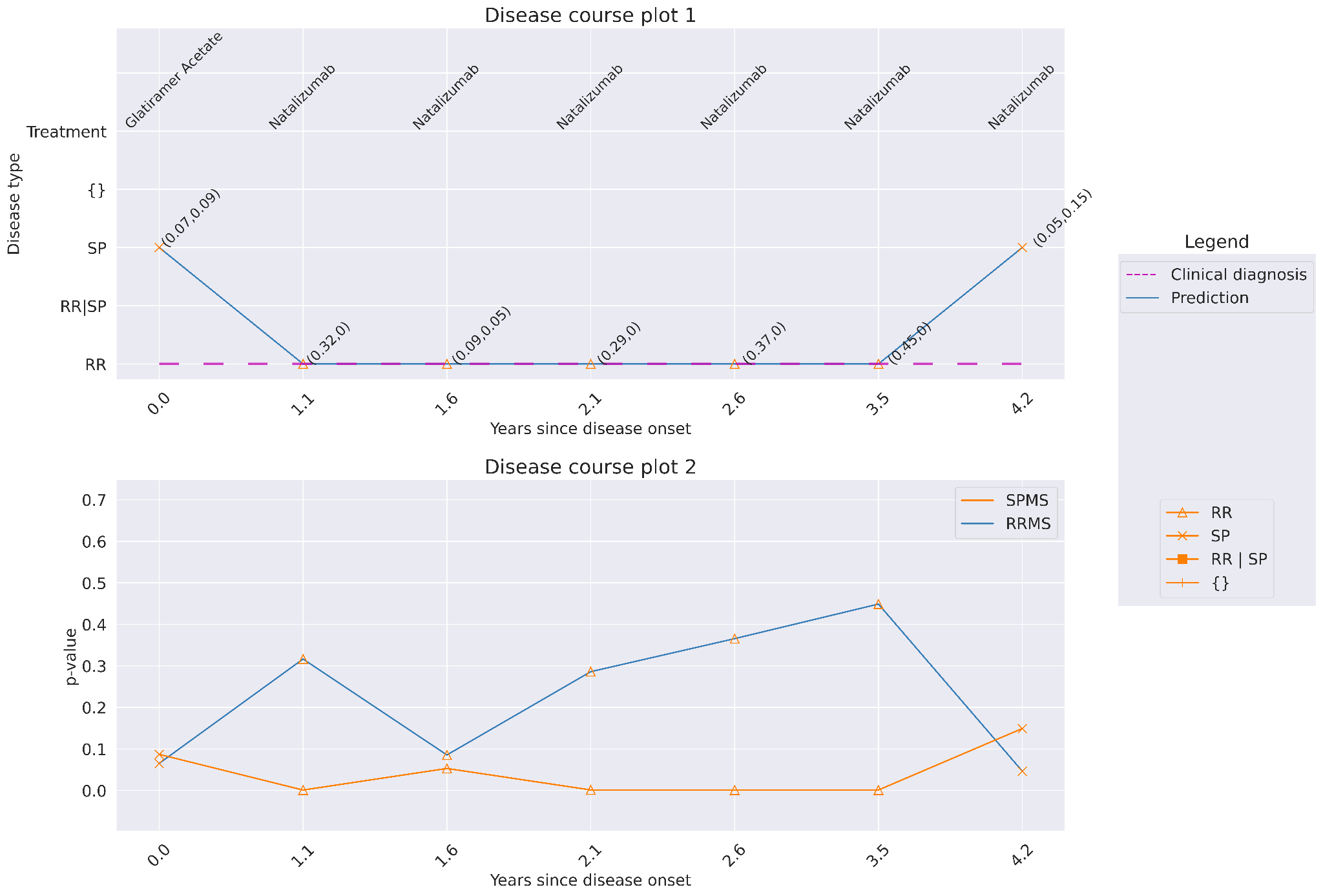


**Supplementary Figure 8: Predictions at a confidence of 93% for a patient with a disease course of 4.2 years over 7 hospital visits.** The patient was diagnosed with RRMS at the onset and the latest hospital visit. The model makes predictions as RRMS for the visit years 1.1, 1.6, 2.1, 2.6, and 3.5, while the initial visit has been predicted as SPMS. The disease trajectory does not hold clinical validity, as the disease course cannot go from SPMS to RRMS. The error is likely to have been caused due to lower p-values for both RRMS and SPMS (disease course plot 2), and these errors can be minimized by increasing the confidence of the model.

**Supplementary Table 2: Prediction made by the conformal predictor on transitioning patients.** Predictions were made on 467 patients diagnosed with RRMS at onset hospital visits and SPMS at later hospital visits. The predictions can be divided into five categories based on the predictions of the initial and final hospital visits. (RRMS - SPMS means RRMS at the initial hospital visit and SPMS at the latest available hospital visit). Np=number of individual patients, nv=number of hospital visits. *Percentages would not add up due to rounding off.

| Predictions  (at the first and latest hospital visit) | Diagnosis of transitioning patients at debut and at latest hospital visit. This is a subset of the SPMS patients from Table 2 (np=467, nv=5,821). |
| --- | --- |
| RRMS - SPMS | 320 (68.5%) |
| SPMS - SPMS | 125 (26.8%) |
| RRMS - RRMS | 21 (4.4%) |
| SPMS - RRMS | 1 (0.2%) |
| RRMS - Multiple-label | 0 (0.0%) |


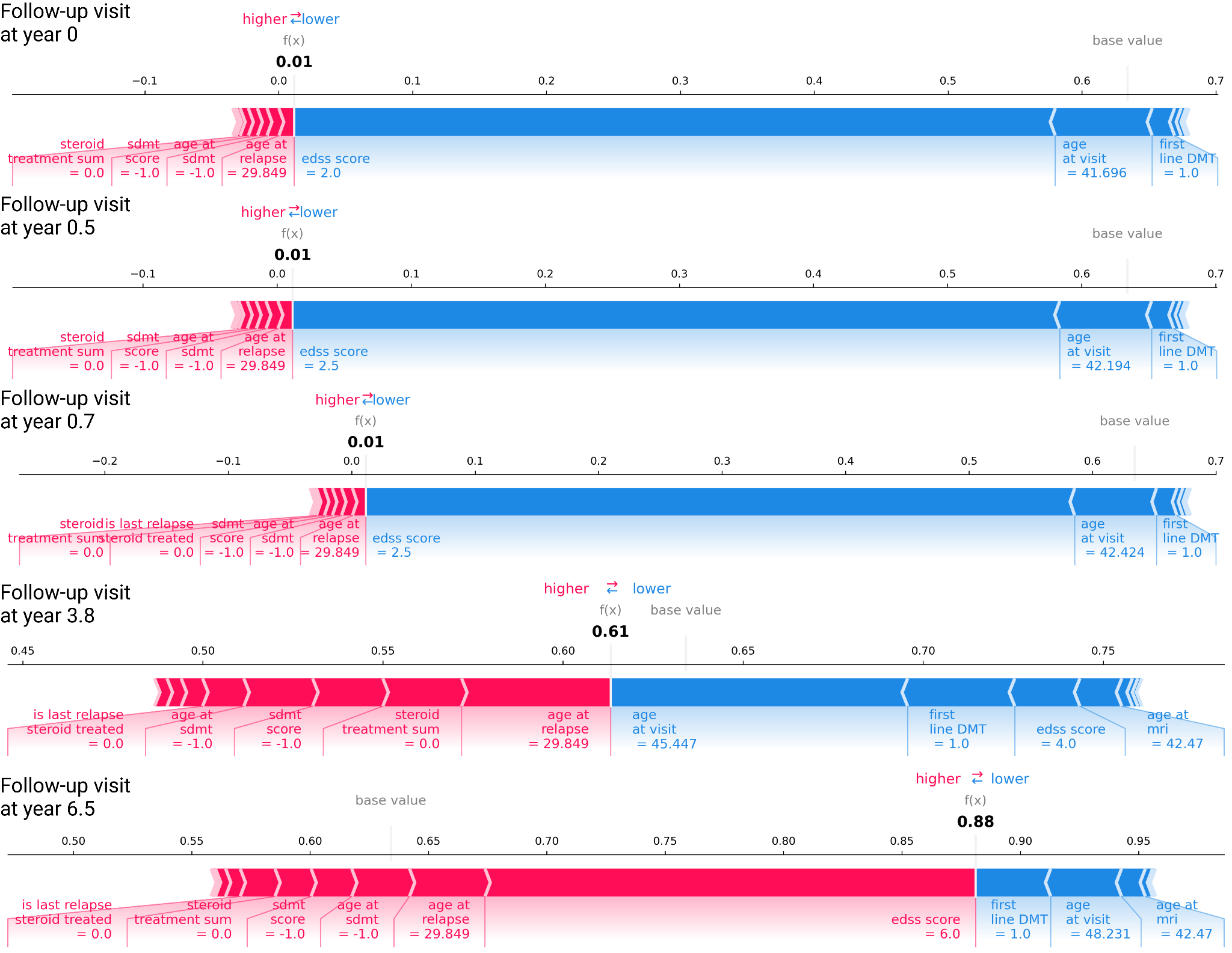


**Supplementary Figure 9: Feature contribution explanation using force plots for the predictions on the hospital visit year 0, 0.5, 0.7, 3.8, and 6.5 on the patient from Figure 6.** During the hospital visit at years 0, 0.5 and 0.7, the patient was predicted to have RRMS with EDSS as a major driving factor. At year 3.8 and 6.5 the patient was predicted with SPMS. EDSS of 6 was the major driving factor for SPMS at year 6.5. The features in red contribute to SPMS and the features in blue contribute to RRMS.


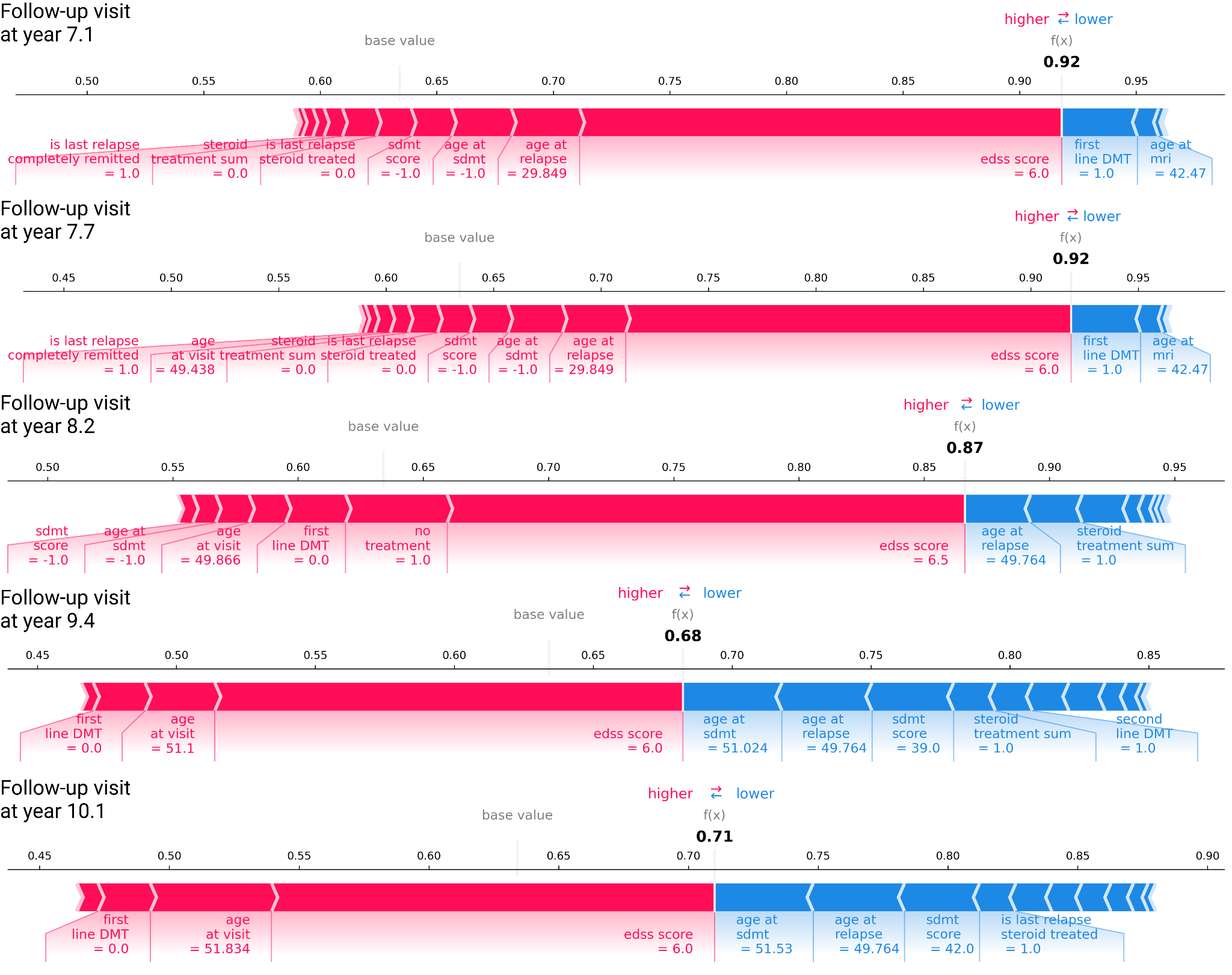


**Supplementary Figure 10: Feature contribution explanation using force plots for the predictions on the hospital visit years 7.1, 7.7, 8.2, 9.4, and 10.1 on the patient from Figure 6.** The patient was predicted with SPMS at all the visits with EDSS as the major driving factor. The features in red contribute to SPMS and the features in blue contribute to RRMS.


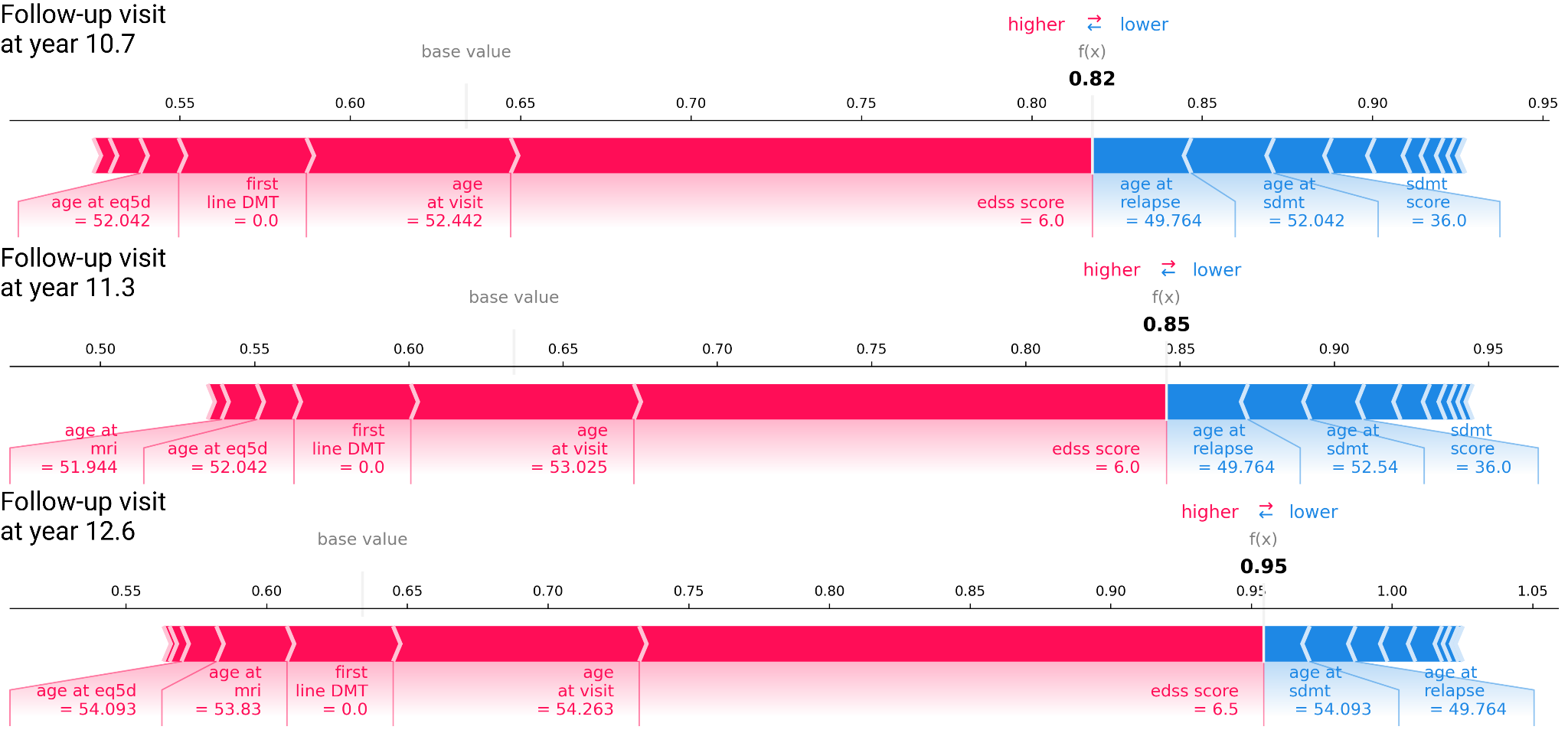


**Supplementary Figure 11: Feature contribution explanation using force plots for the predictions on the hospital visit years 10.7, 11.3, and 12.6 on the patient from Figure 6.** The patient was predicted with SPMS at all the visits with EDSS as the major driving factor. The features in red contribute to SPMS and the features in blue contribute to RRMS.


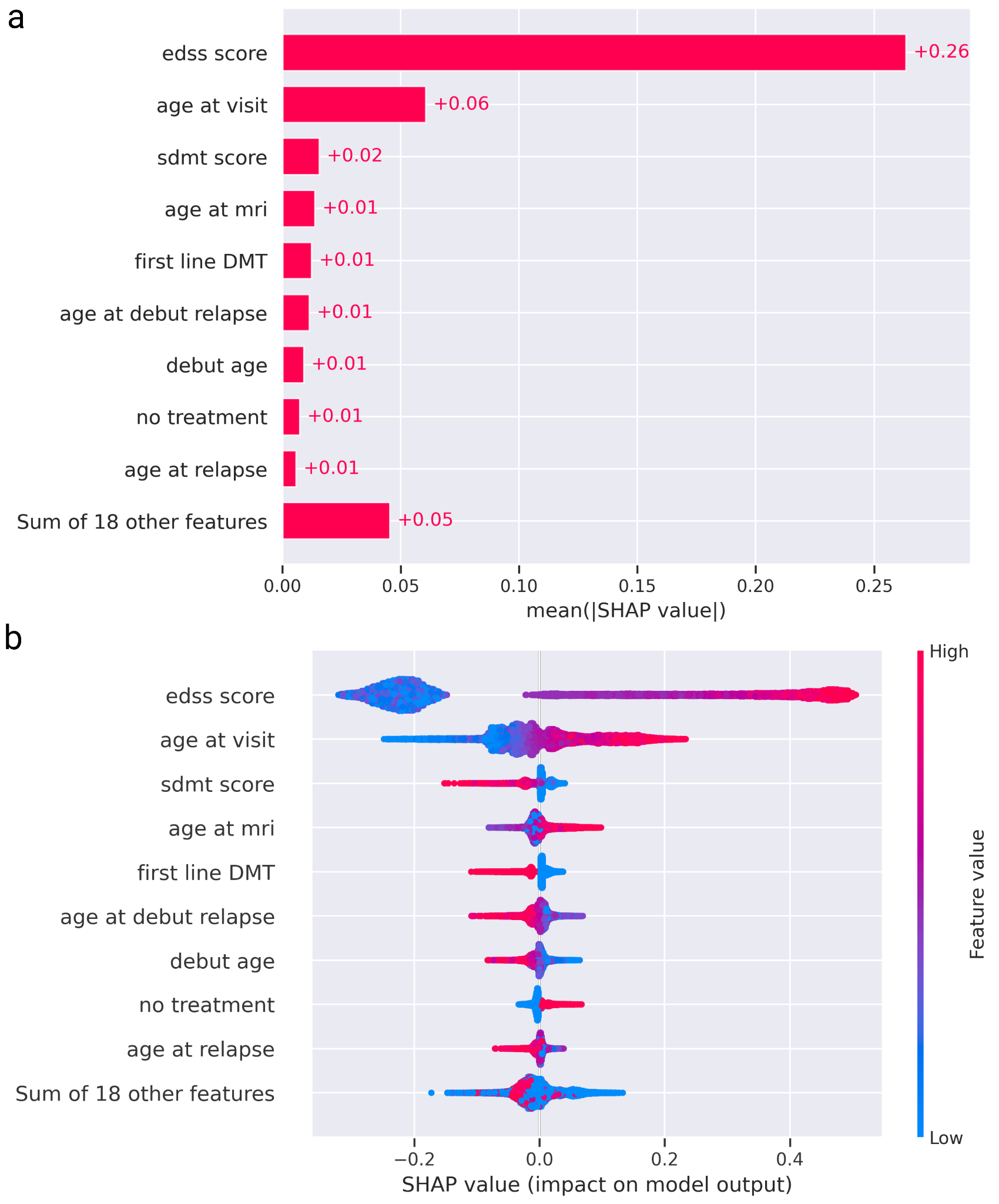


**Supplementary Figure 12: The global importance of features for prediction on the test data is explained using SHAP.** (a) a summary plot using mean absolute SHAP values, (b) a bee swarm plot showing the SHAP values for each feature, calculated iteratively for each datapoint in the dataset. The values are plotted and color-coded based on their importance for each prediction.

# Predictions for six patients with higher uncertainty, with confidence levels of 93%, 95%, and 99%.

#

# **
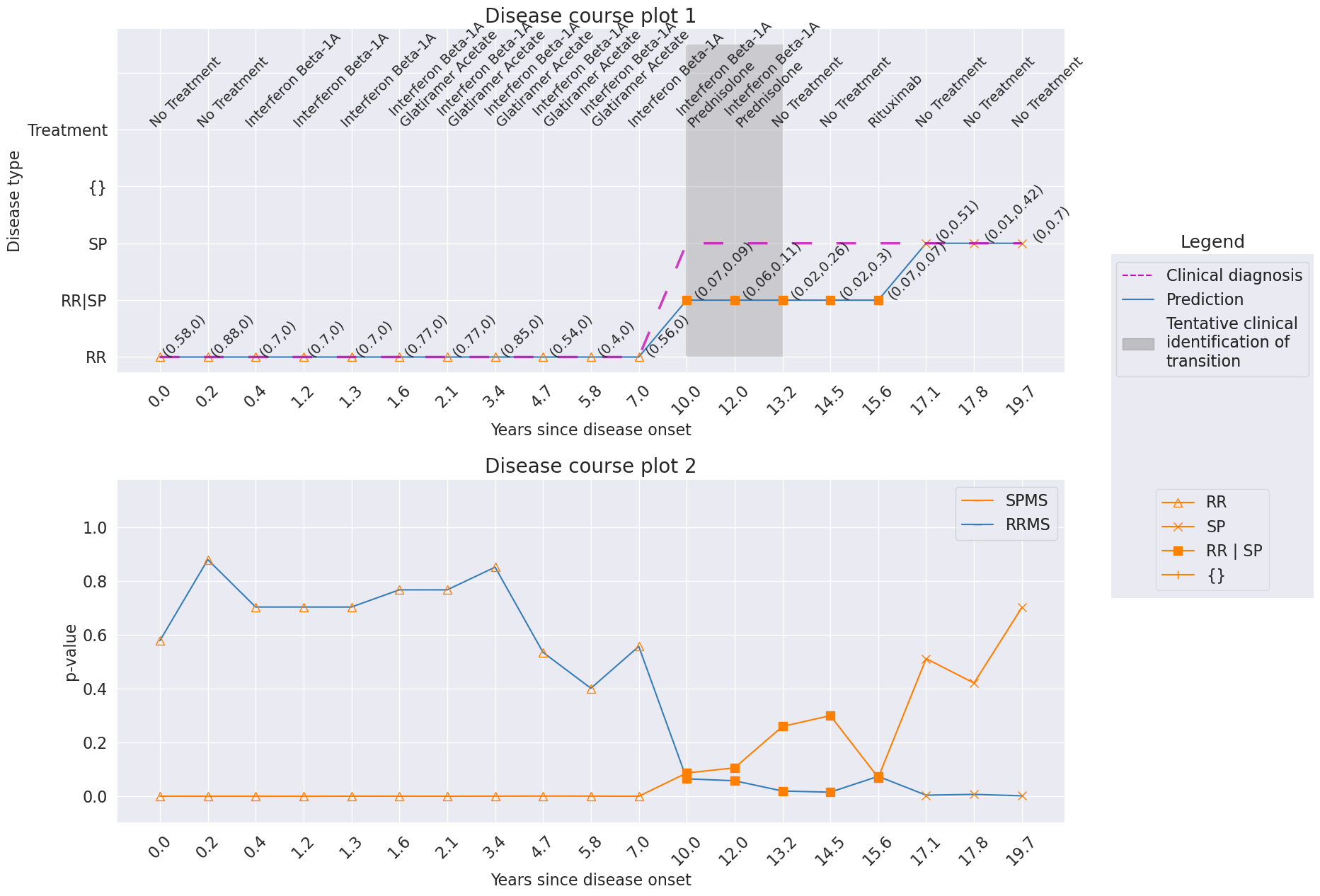
**

**Supplementary Figure 13: Patient 1. Predictions at a confidence of 99% for a patient with a disease course of 19.7 years over 19 hospital visits.** There are more unreliable (multiple) predictions between the years 10.0 and 15.6. The predictions for years 13.2 and 14.5 became unreliable when the confidence increased from 95% to 99%.


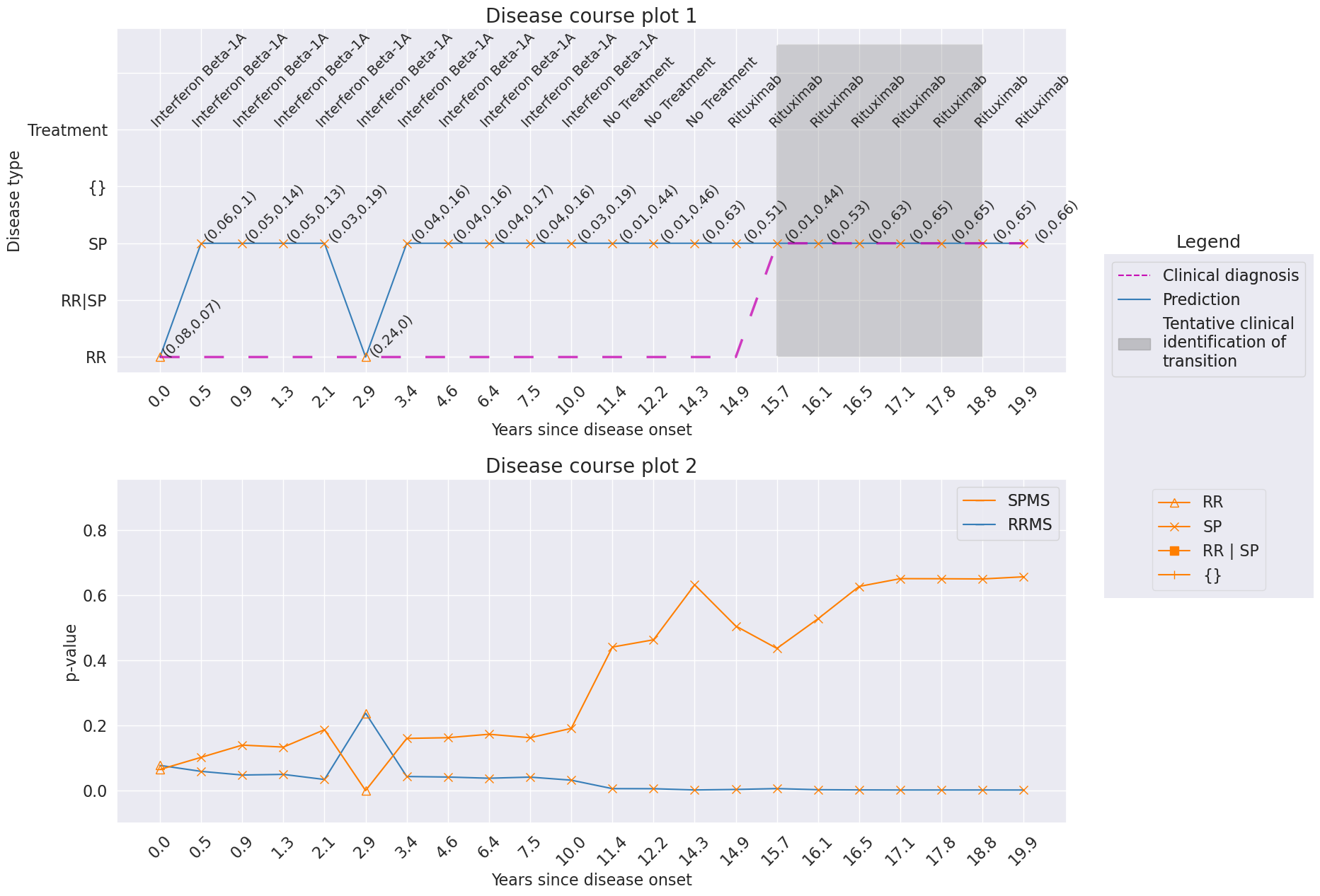


**Supplementary Figure 14: Patient 2. Predictions at a confidence of 93% for a patient with a disease course of 19.9 years over 22 hospital visits.** According to the predictions, the patient switched disease state from SPMS to RRMS at year 2.9 and back to SPMS at year 3.4, which is clinically impossible.


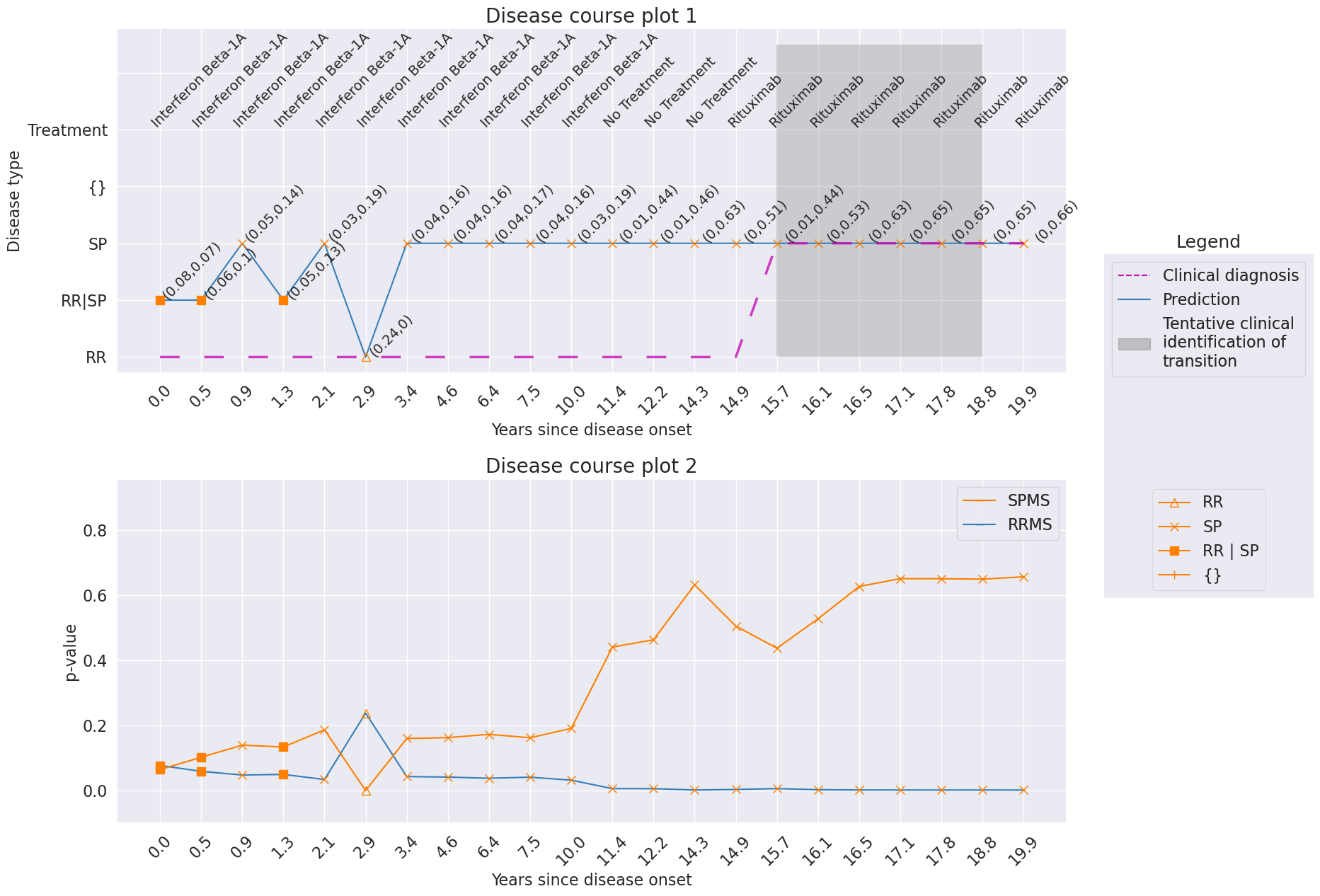


**Supplementary Figure 15: Patient 2. Predictions at a confidence of 95% for a patient with a disease course of 19.9 years over 22 hospital visits.** The disease trajectory still does not hold clinical validity as the disease state switched from SPMS to RRMS at year 2.9 and back to SPMS at year 3.4.


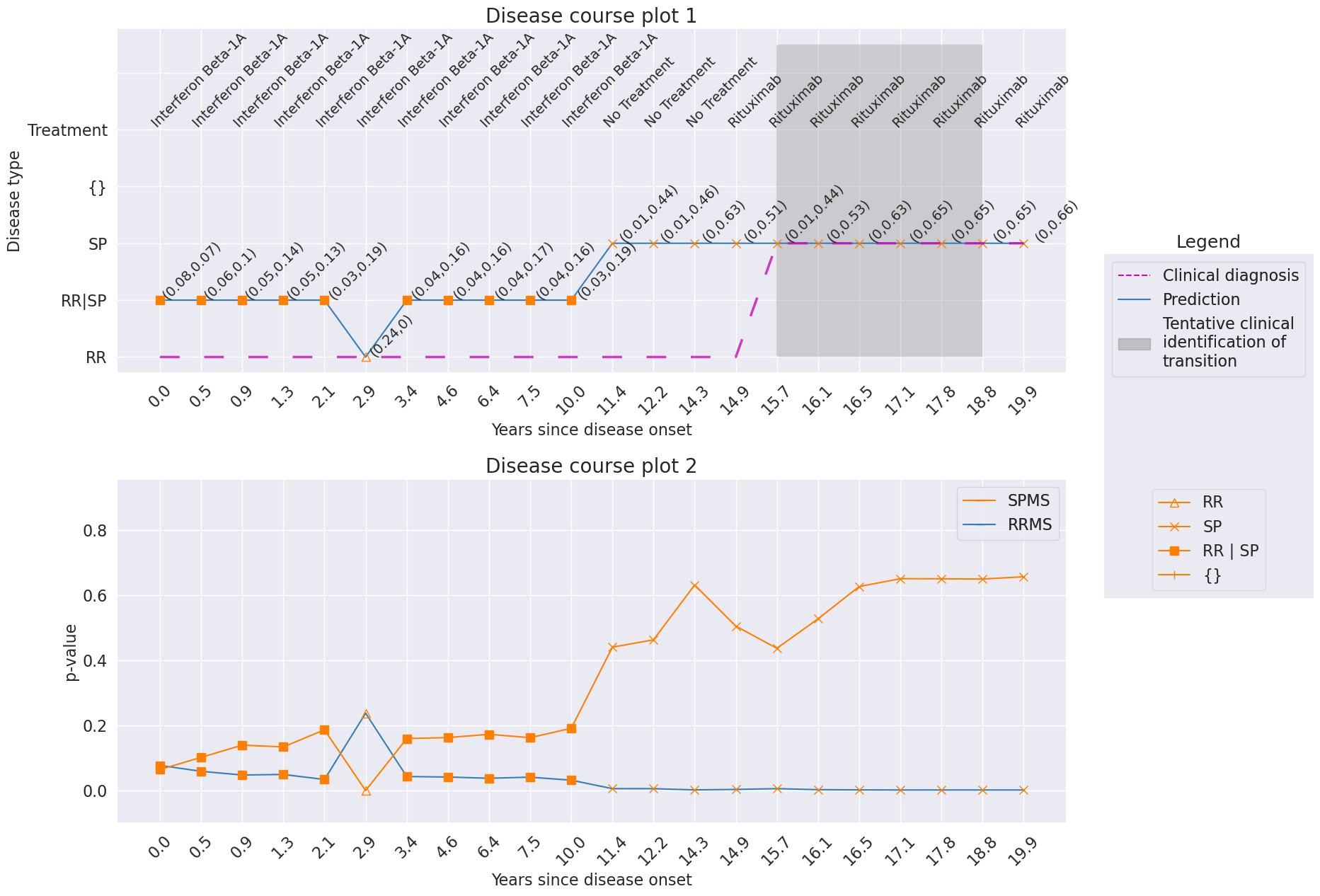


**Supplementary Figure 16:** **Patient 2. Predictions at a confidence of 99% for a patient with a disease course of 19.9 years over 22 hospital visits.** There is an increase in multiple-label, and the predicted SPMS transition is at year 11.4, while the clinical transition was marked at year 15.7 in the clinic. The disease trajectory is now clinically valid.


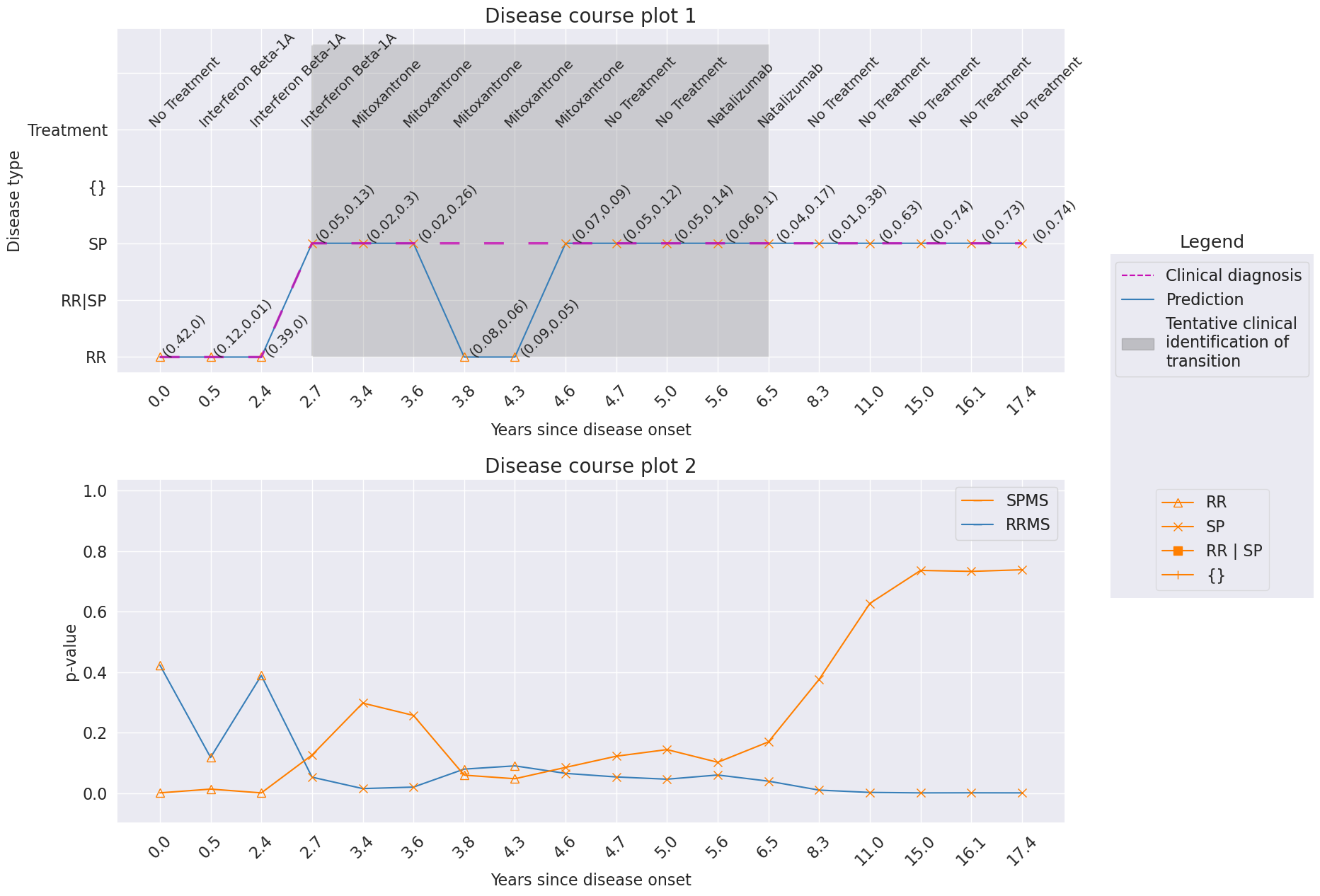


**Supplementary Figure 17: Patient 3. Predictions at a confidence of 93% for a patient with a disease course of 17.4 years over 18 hospital visits.** The patient switched from SPMS to RRMS at year 3.8, while changing back to SPMS again at year 4.6. The alternating prediction can be associated with lower p-values of these predictions (Disease course plot 2).


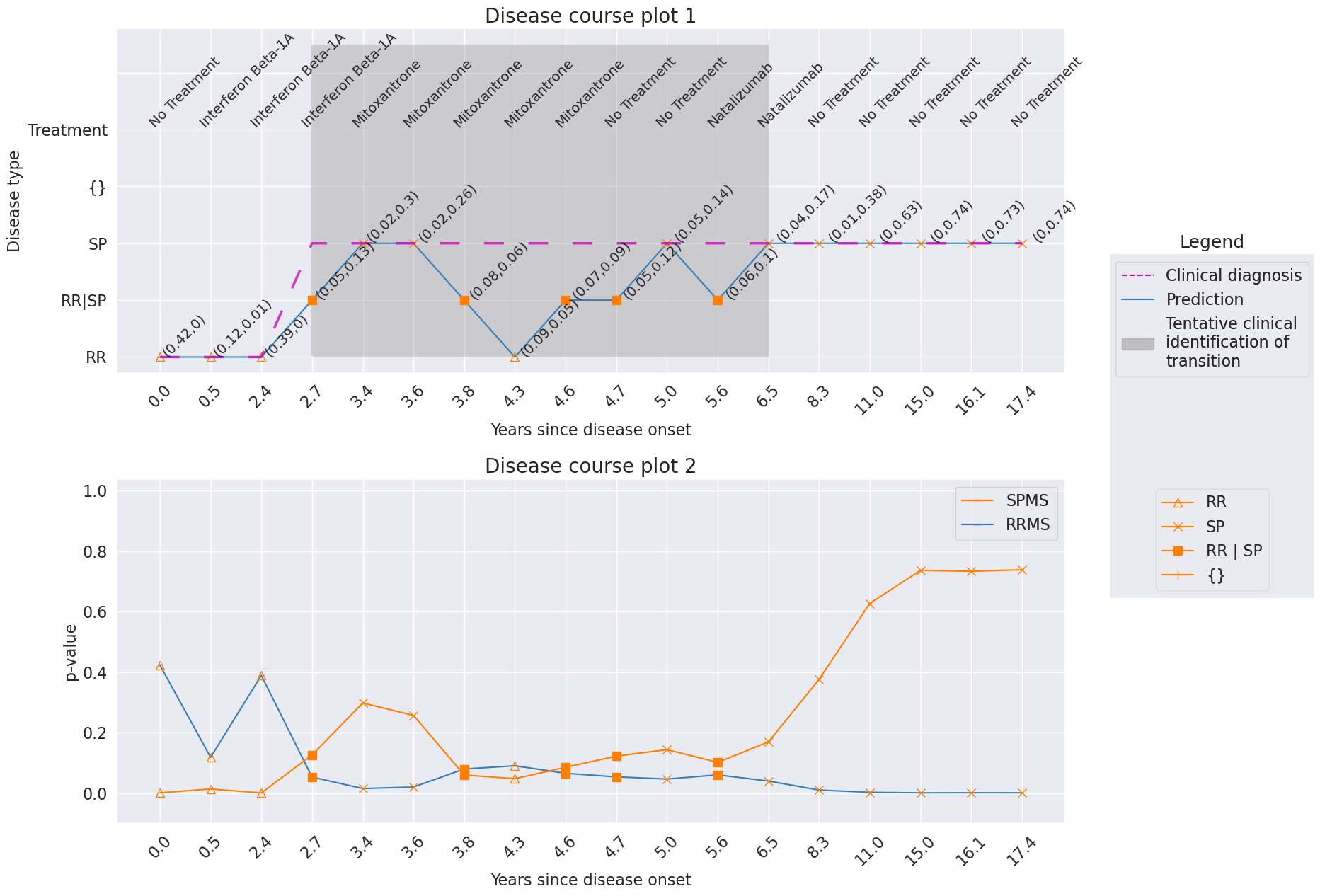


**Supplementary Figure 18: Patient 3. Predictions at a confidence of 95% for a patient with a disease course of 17.4 years over 18 hospital visits.** The lower p-value predictions became multiple-label, yet the disease trajectory remained clinically invalid.


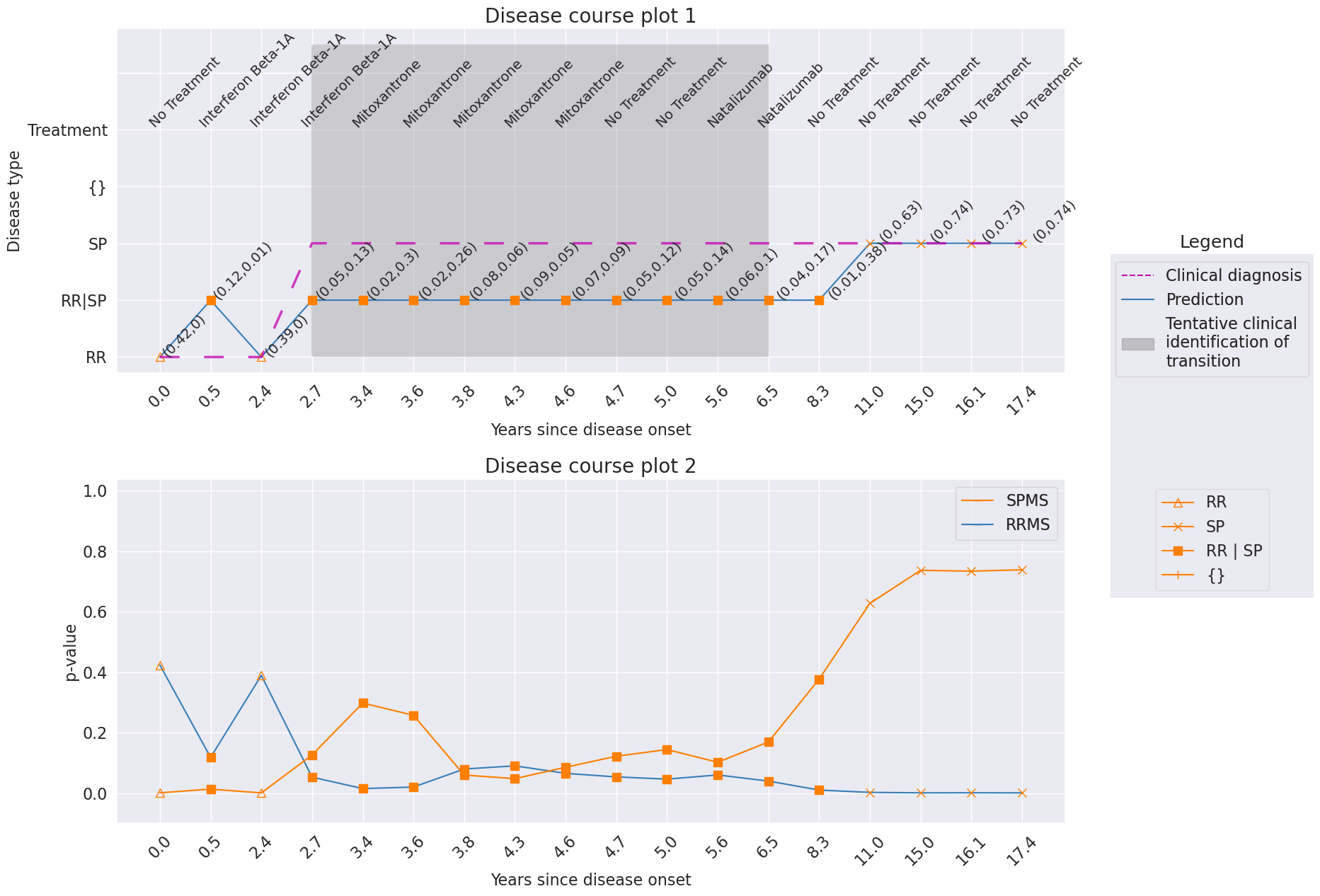


**Supplementary Figure 19: Patient 3. Predictions at a confidence of 99% for a patient with a disease course of 17.4 years over 18 hospital visits.** The predictions became more stringent, marking the predictions between years 2.7 to 8.3 multiple-label. The model predicts transition at the year 11.0 at this confidence.


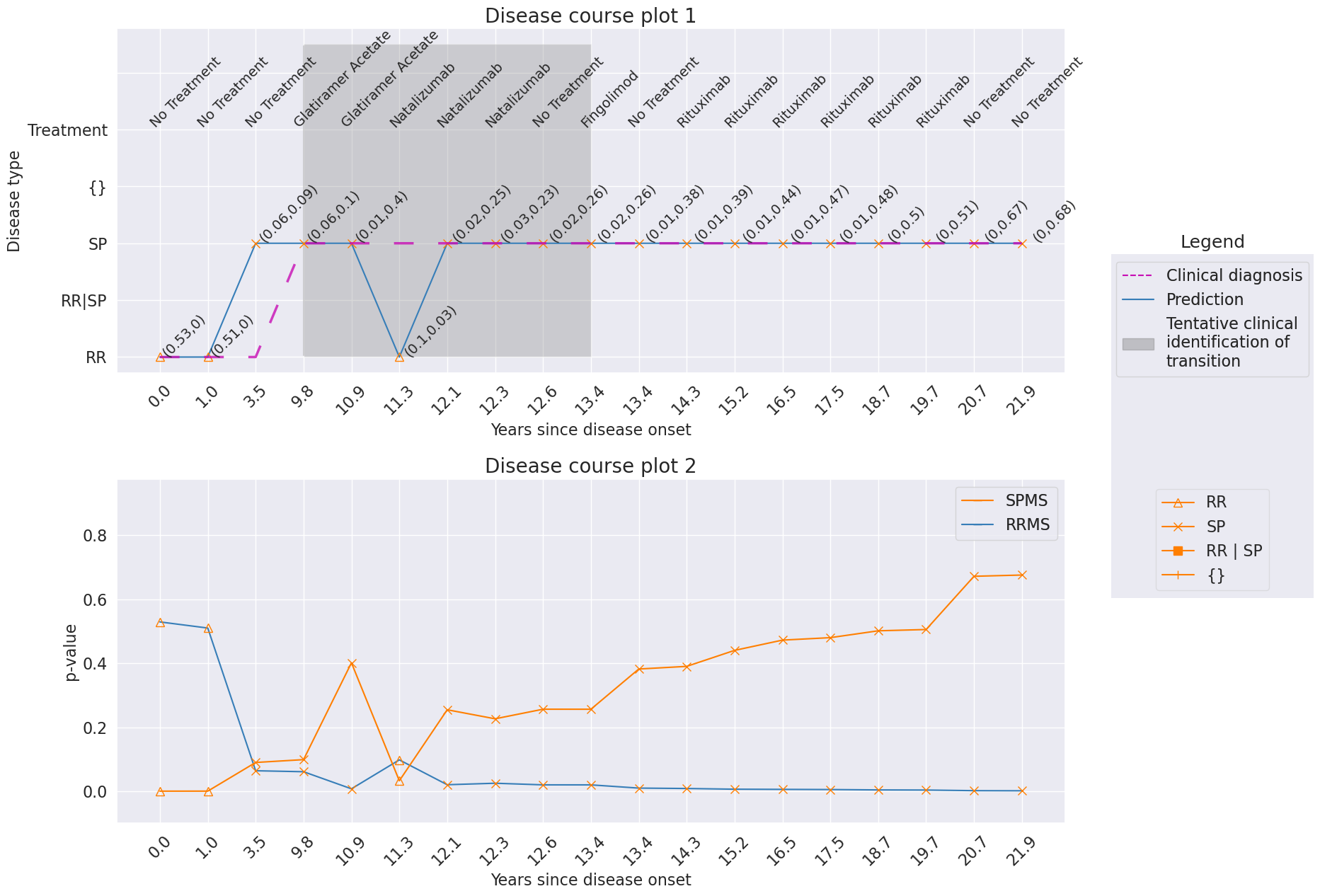


**Supplementary Figure 20: Patient 4. Predictions at a confidence of 93% for a patient with a disease course of 21.9 years over 19 hospital visits.** The disease trajectory is invalid as the disease transitioned from SPMS to RRMS at visit year 11.3.


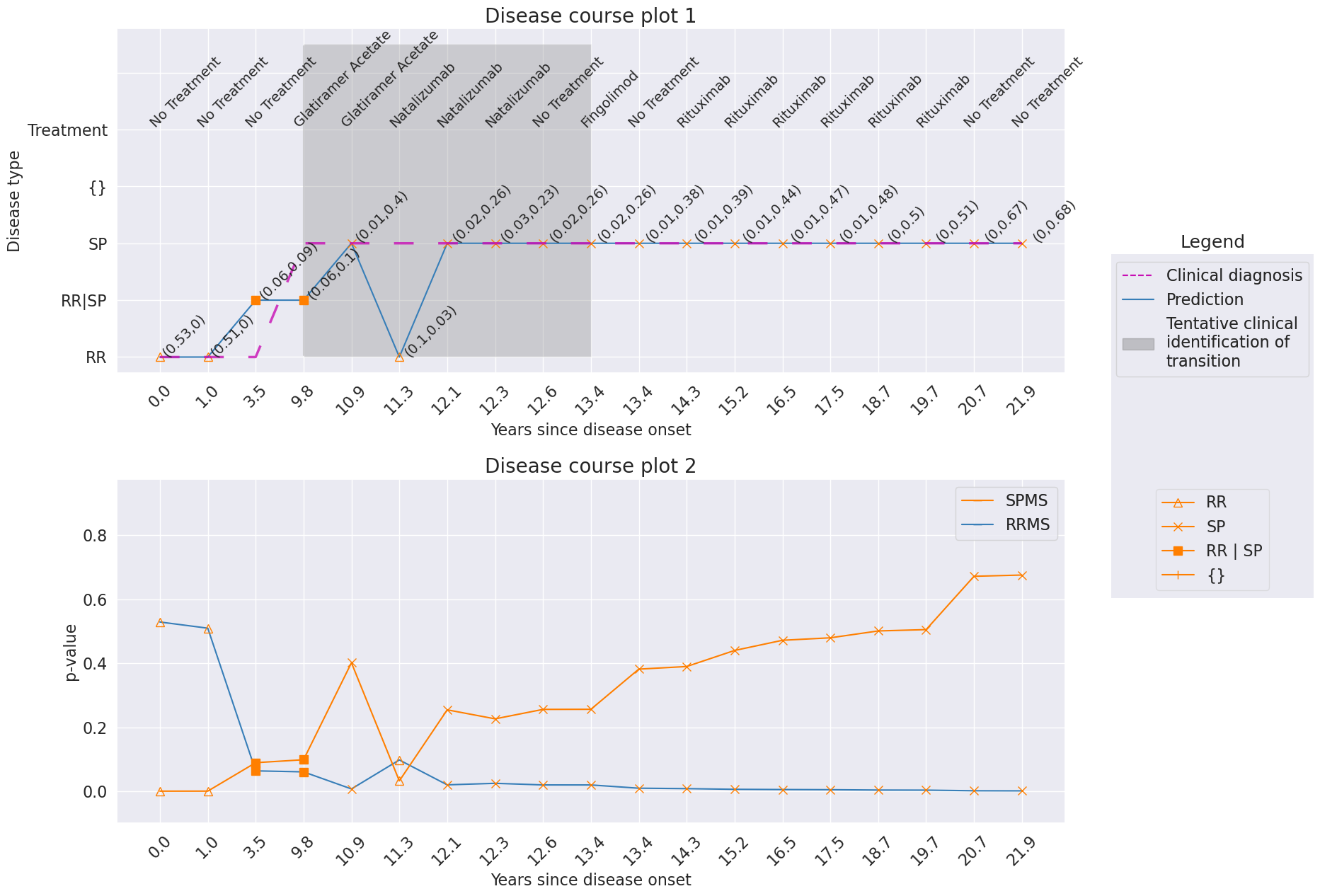


**Supplementary Figure 21: Patient 4. Predictions at a confidence of 95% for a patient with a disease course of 21.9 years over 19 hospital visits.** The predictions at years 3.5 and 9.8 became multiple-label with increased prediction confidence. However, the disease trajectory remains clinically invalid because the disease state changes from SPMS to RRMS at year 11.3.


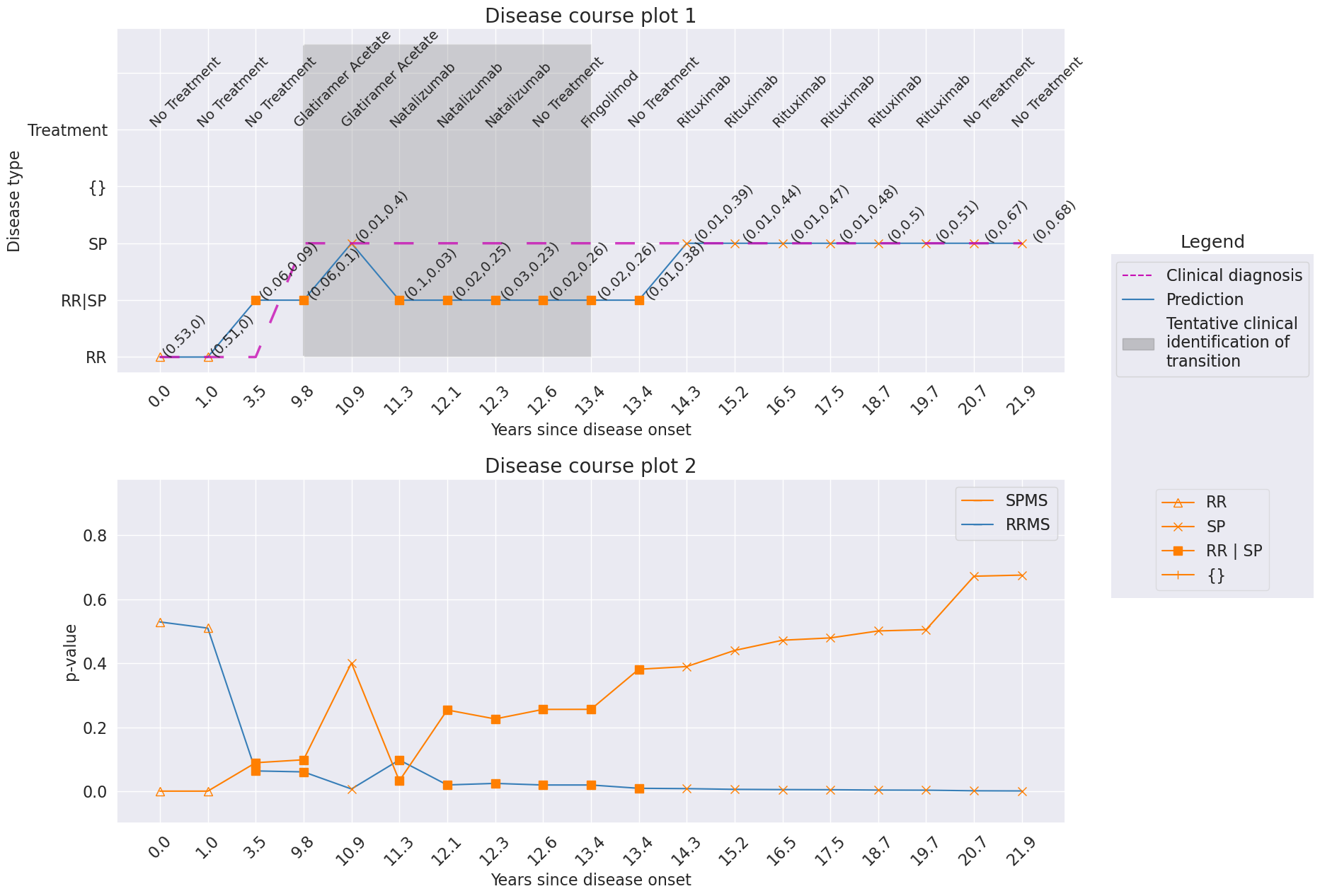


**Supplementary Figure 22:** **Patient 4. Predictions at a confidence of 99% for a patient with a disease course of 21.9 years over 19 hospital visits.** With further increase in confidence, the model predicted transition at year 10.9, with a valid disease trajectory.


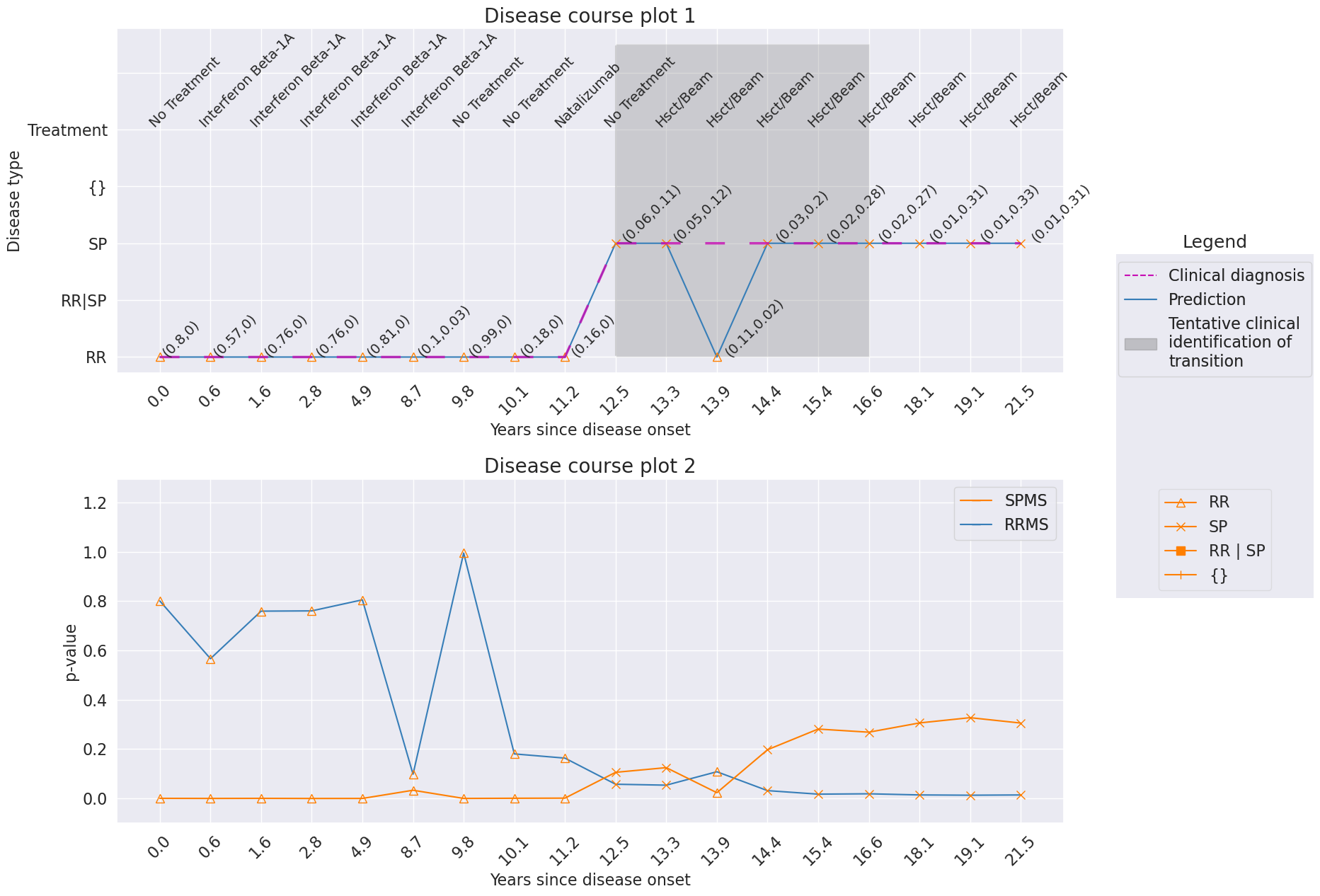


**Supplementary Figure 23: Patient 5. Predictions at a confidence of 93% for a patient with a disease course of 21.5 years and having 18 hospital visits.** The disease trajectory is not clinically valid as the model predicts the patient’s disease state to change from SPMS to RRMS at year 13.9.


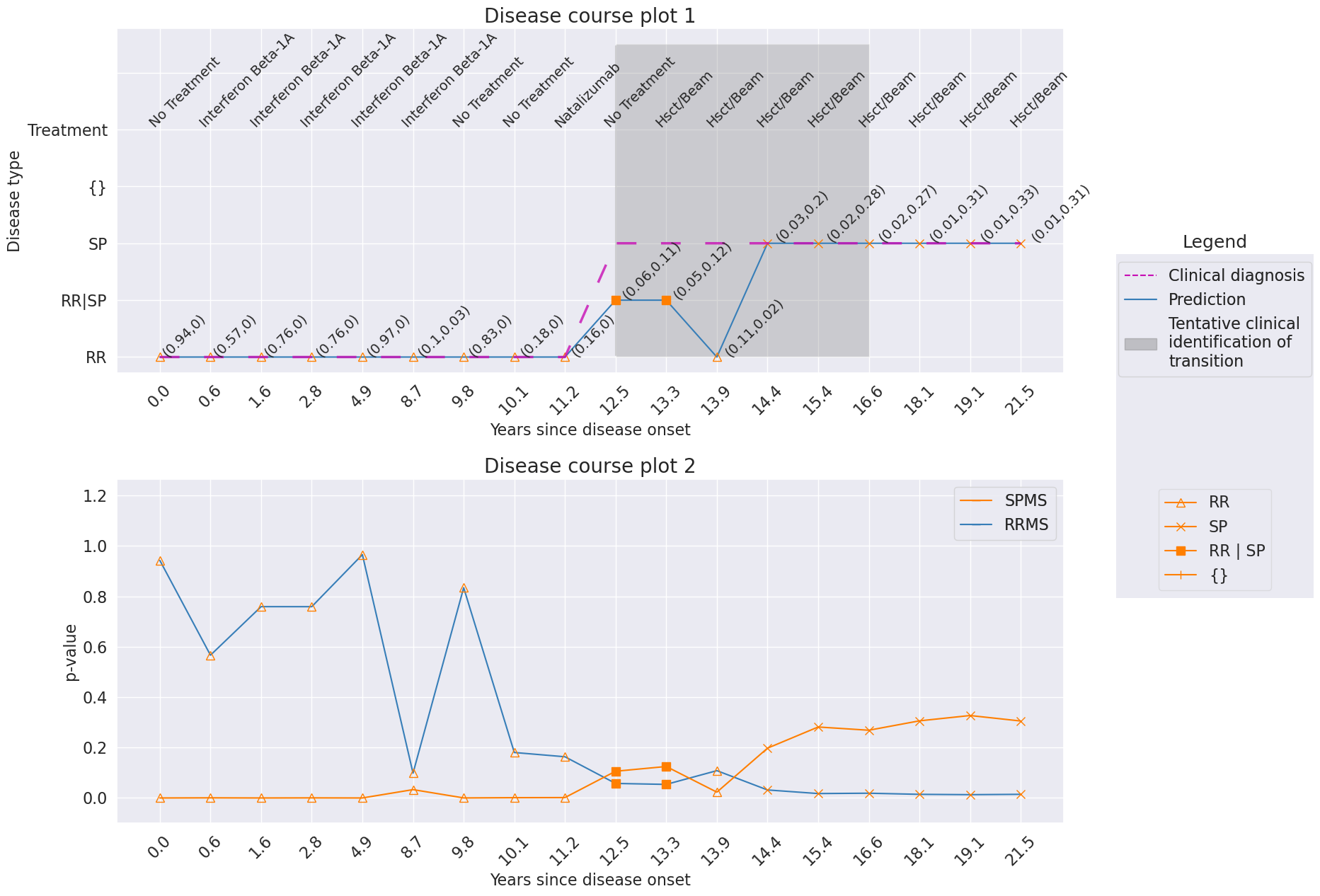


**Supplementary Figure 24: Patient 5. Predictions at a confidence of 95% for a patient with a disease course of 21.5 years over 18 hospital visits.** The model produced a clinically valid disease trajectory as the confidence increased, with the transition marked at year 14.4.


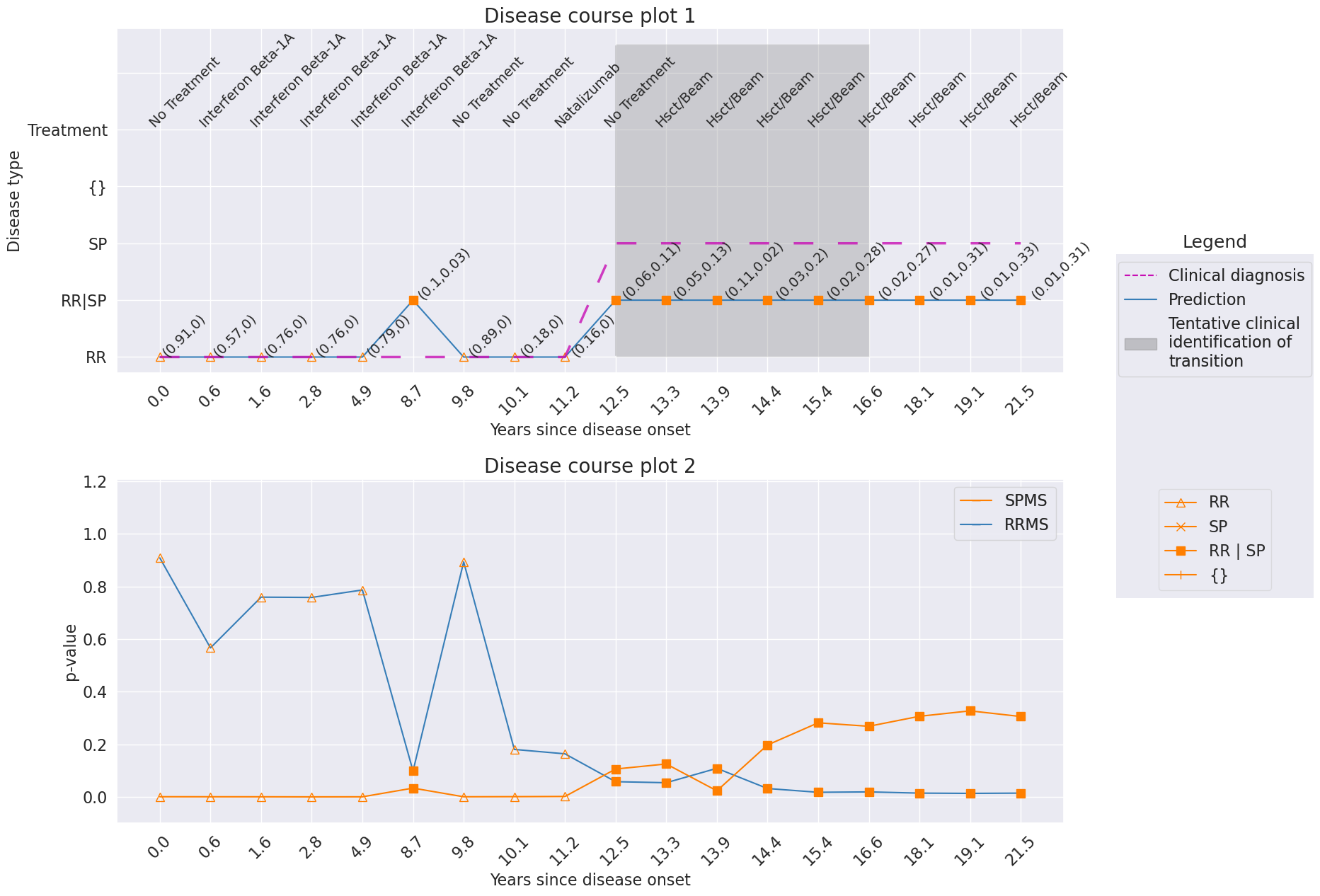


**Supplementary Figure 25: Patient 5. Predictions at a confidence of 99% for a patient with a disease course of 21.5 years over 18 hospital visits.** Though there is a higher p-value for SPMS from visit 14.4 and onwards, the model does not predict single-label for these hospital visits at this high confidence.


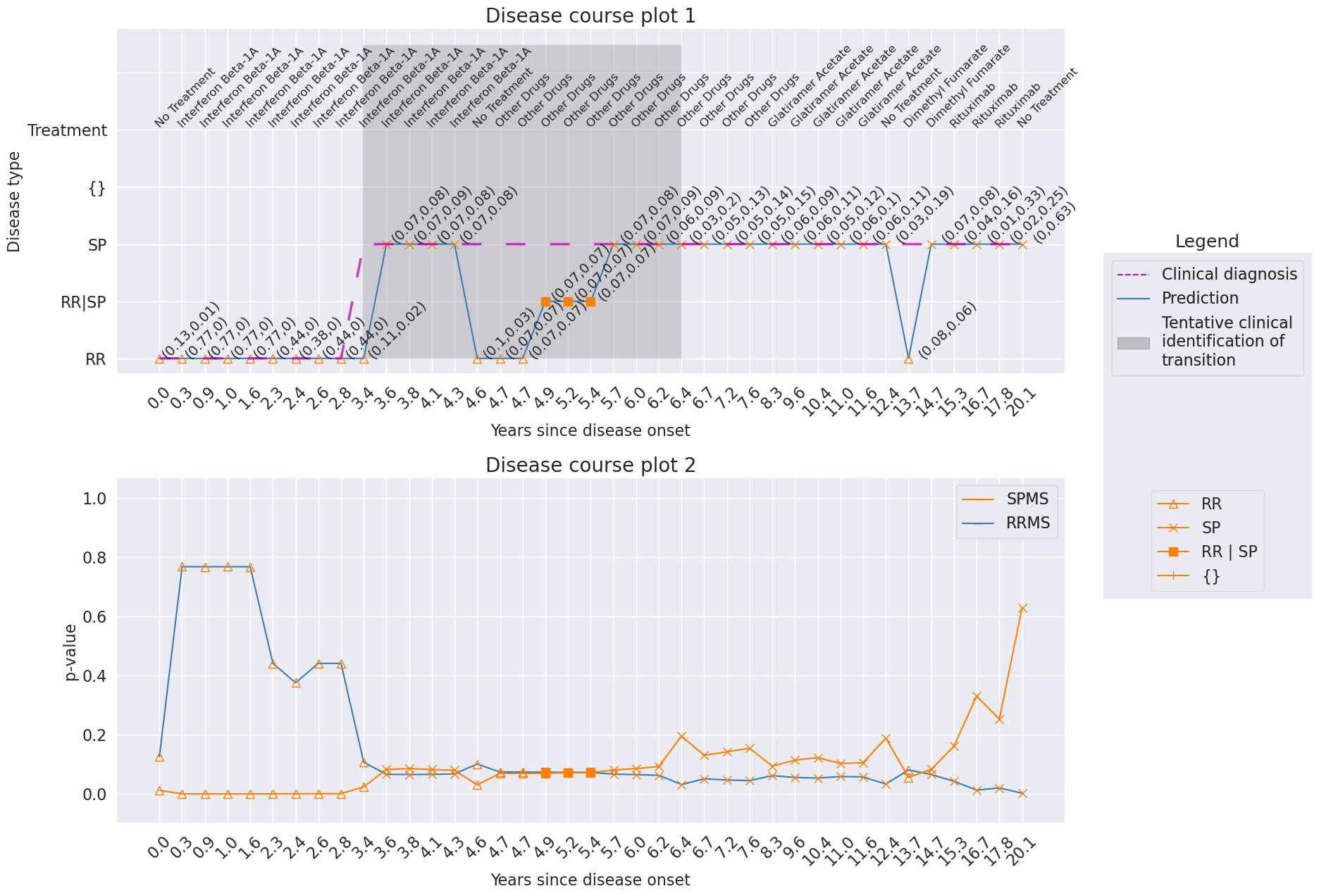


**Supplementary Figure 26: Patient 6. Predictions at a confidence of 93% for a patient with a disease course of 20.1 years over 39 hospital visits.** The predictions changes to RRMS from SPMS at year 4.6 and year 13.7 produces a clinically invalid disease trajectory.


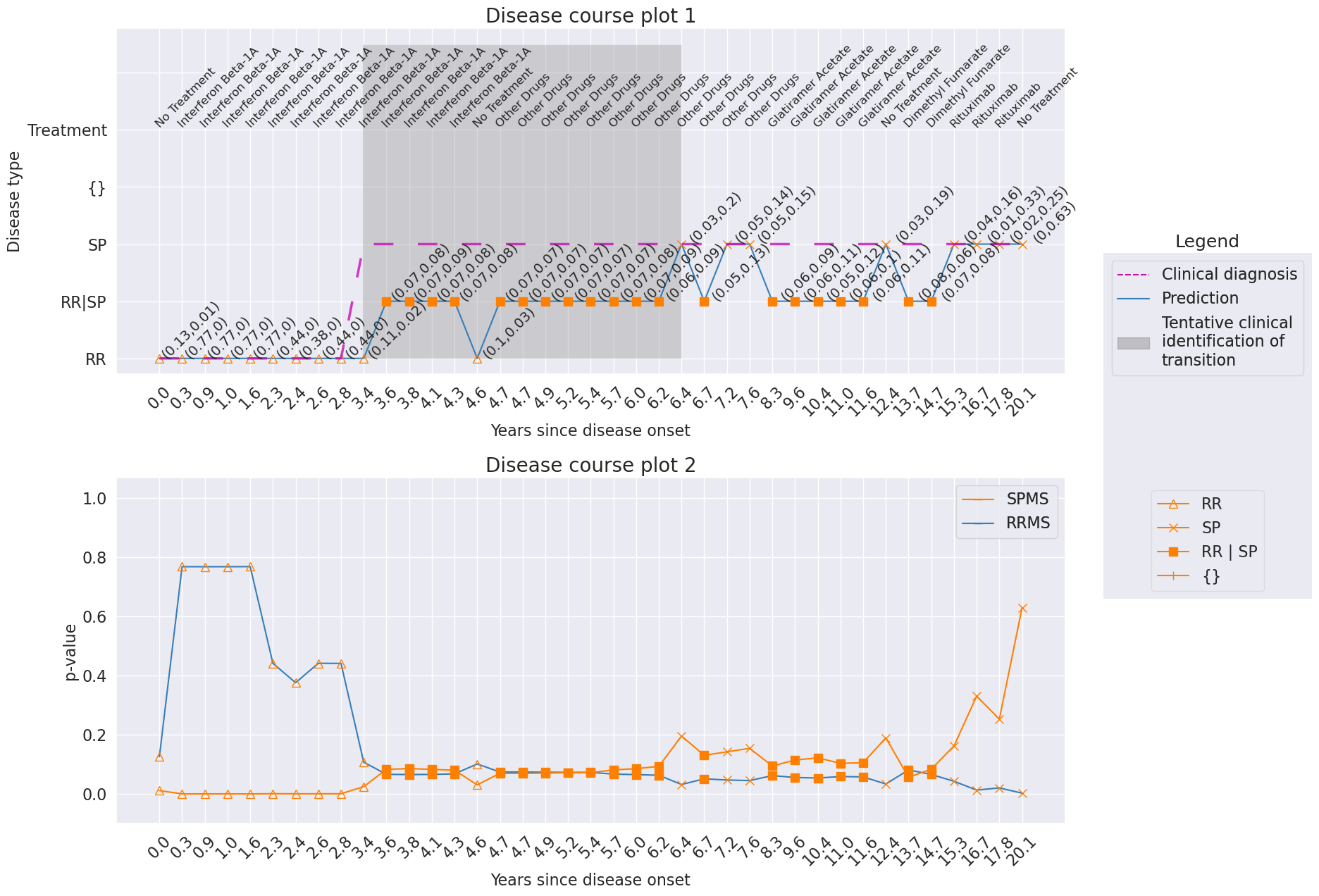


**Supplementary Figure 27: Patient 6. Predictions at a confidence of 95% for a patient with a disease course of 20.1 years over 39 hospital visits.** The disease trajectory now holds clinical validity, and there is an increase in multiple-label predictions. The p-values for these predictions are low, indicating the patient could be in an extended transition phase.


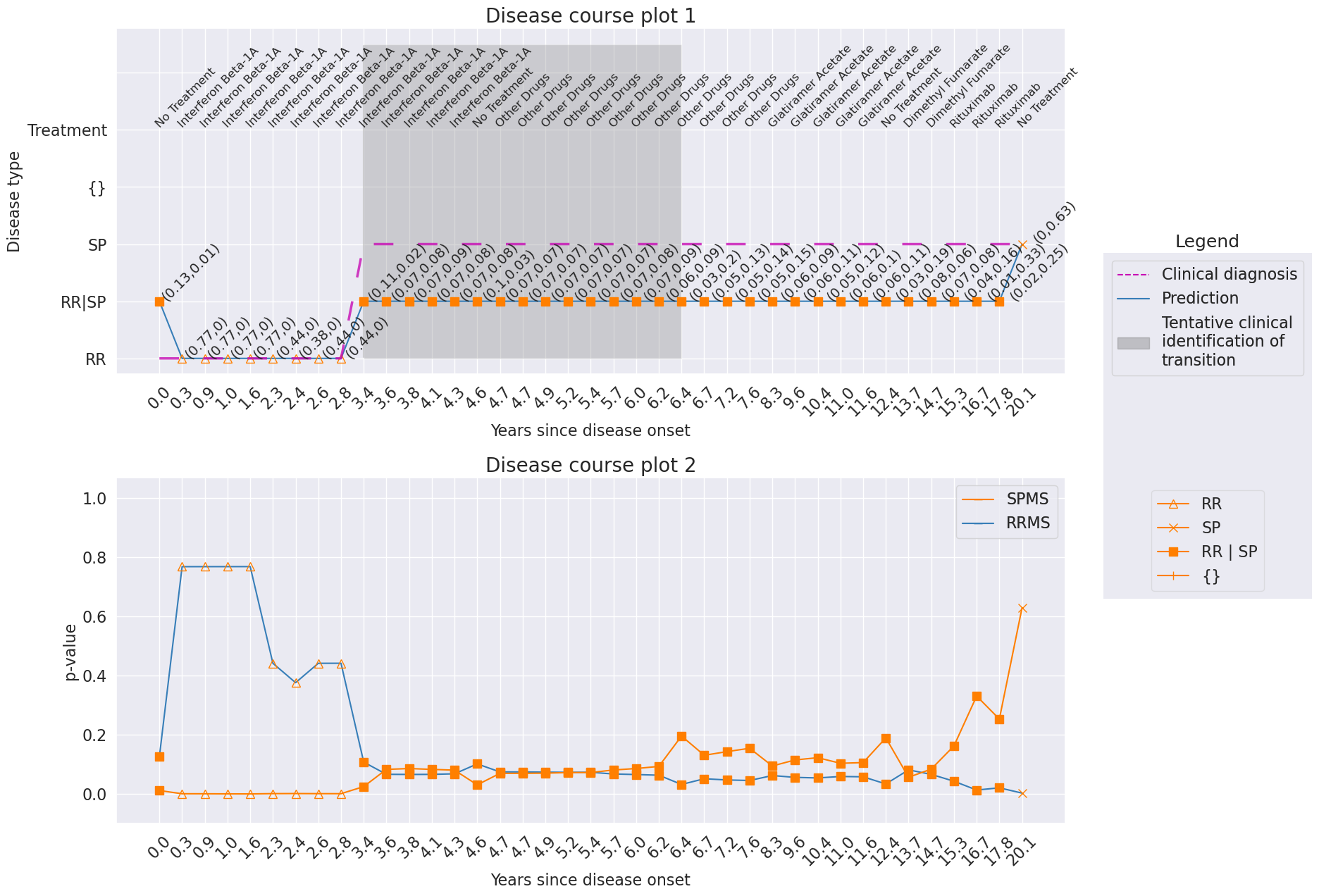


**Supplementary Figure 28: Patient 6. Predictions at a confidence of 99% for a patient with a disease course of 20.1 years over 39 hospital visits.** On further increase in confidence level, there is an increase in multiple label prediction. These predictions indicate an extended transition period for a patient between year 3.4 and year 17.8.

# Supplementary references

1. [Hobart, J., Lamping, D., Fitzpatrick, R., Riazi, A. & Thompson, A. The Multiple Sclerosis Impact Scale (MSIS-29): a new patient-based outcome measure. *Brain* **124**, 962–973 (2001).](http://paperpile.com/b/useNBn/sNib)
